# Cardiomyocyte-derived USP28 negatively regulates antioxidant response and promotes cardiac hypertrophy via deubiquitinating TRIM21

**DOI:** 10.7150/thno.99340

**Published:** 2024-09-30

**Authors:** Jibo Han, Liming Lin, Zimin Fang, Bozhi Ye, Xue Han, Jiachen Xu, Binjiang Han, Julian Min, Jinfu Qian, Gaojun Wu, Yi Wang, Guang Liang

**Affiliations:** 1 Department of Cardiology, the First Affiliated Hospital of Wenzhou Medical University, Wenzhou, Zhejiang, China.; 2 Chemical Biology Research Center, School of Pharmaceutical Sciences, Wenzhou Medical University, Wenzhou, Zhejiang, China.; 3 Department of Cardiology, the Second Affiliated Hospital of Jiaxing University, Jiaxing, Zhejiang, China.; 4 Department of Pharmacy and School of Pharmaceutical Sciences, Zhejiang Provincial People's Hospital, Affiliated People's Hospital, Hangzhou Medical College, Hangzhou, Zhejiang, 310014, China.; 5 School of Pharmaceutical Sciences, Hangzhou Normal University, Hangzhou, Zhejiang, China.

**Keywords:** Deubiquitinating enzyme, USP28, Cardiomyocyte, Cardiac hypertrophy, TRIM21.

## Abstract

**Rationale:** Cardiac hypertrophy is an important pathological basis for heart failure. Most physiological activities of cardiomyocytes are regulated by proteins and their post-translational modification. Deubiquitinating enzymes (DUBs) are involved in protein stability maintenance and closely related to myocardial hypertrophy. In this study, we aimed to clarify the regulatory role of a DUB, ubiquitin-specific peptidase 28 (USP28), in cardiac hypertrophy and explore the molecular mechanism behind.

**Methods:** Transcriptome and single-cell mRNA sequencing was used to demonstrate the association of USP28 and cardiac hypertrophy. Cardiomyocyte-specific USP28 knockout mice (USP28CKO) were subjected to angiotensin II (Ang II) infusion or transverse aortic constriction (TAC) models. Coimmunoprecipitation combined mass spectrum analysis (Co-IP/MS) was applied to screen out the substrate of USP28.

**Results:** We first showed the up-regulation of USP28 in cardiac hypertrophy, and its cellular localization of cardiomyocytes. USP28CKO protects mouse heart against Ang II- or TAC-induced cardiac dysfunction and hypertrophy. Mechanistically, we identified tripartite motif-containing protein 21 (TRIM21) as the potential substrate of USP28 by Co-IP/MS analysis. Cardiomyocyte USP28 deubiquitinates and stabilizes TRIM21 to negatively regulate nuclear factor erythroid 2-related factor 2 (Nrf2) antioxidant response, increasing oxidative stress in cardiomyocytes and promoting cardiac hypertrophy and injury. Finally, using a selective USP28 inhibitor Otilonium Bromide, we confirmed the therapeutic effect of pharmacological inhibition of USP28 against TAC-induced established hypertrophic heart failure.

**Conclusion:** Our study illustrates a cardiomyocyte-specific USP28-TRIM21 axis in regulating hypertrophic cardiomyopathy and presents USP28 as a potential target for the treatment of cardiac hypertrophy.

## Introduction

Chronic heart failure (HF) has become the leading cause of hospitalization for people over 65 years old 1, 2. Cardiomyocyte hypertrophy is an important pathological basis for the occurrence and development of HF and an independent risk factor for a variety of cardiovascular diseases (CVDs) 3. As myocardial hypertrophy progresses, patients may experience reduced cardiac volume and develop adverse cardiovascular events such as sudden cardiac death 3. Although pharmacological and instrumental treatment of HF has advanced in recent years, the prognosis and quality of patients are still poor. Discovery of new therapies against HF needs more understanding on the pathological actions of hypertrophic cardiomyocytes 1, 2.

Most pathophysiological actions of cells are regulated by proteins, which depend not only on protein expression but also on protein post-translational modification (PTM) 4. As an important PTM type, ubiquitination can induce substrate protein degradation via ubiquitin-proteasome system (UPS) or affect substrate activity/function 5. As a terminally differentiated cell type, cardiomyocyte has poor regenerative ability 6, therefore, the pathophysiological actions of cardiomyocytes can be regulated by the ubiquitin modification of key intracellular proteins. Ubiquitination is a reversible PTM in which substrate is ubiquitinated by E3 ubiquitin ligase and deubiquitinated by deubiquitinating enzymes (DUBs) 5. DUBs are widely involved in biological processes through deubiquitinating substrates 7. Recent studies have reported that DUBs are involved in the regulation of myocardial hypertrophy 8.

In this study, through transcriptome and single-cell mRNA sequencing (scRNA-seq), we found that a DUB, ubiquitin-specific peptidase 28 (USP28), is involved in cardiac hypertrophy. USP28 was discovered in 2001 9 and the full-length USP28 contain 1077 amino acids, comprising ubiquitin-associated domain (UBA), ubiquitin interacting motif (UIM), and catalytic USP domain harboring the enzymatic active site (C171) 10. USP28 has been reported to be up-regulated in neurodegenerative diseases 11, a variety of tumors 12, and immune system diseases 13, and knockout or inhibition of USP28 can effectively mitigate these diseases 11-13, suggesting the potential of USP28 as a drug target. Especially, USP28 mediates the progression of gastric cancer by inducing intracellular reactive oxygen species (ROS) production 14, indicating that USP28 is an upstream regulator of oxidative stress and may possess potential role in ROS-driven heart diseases. In addition, both non-specific and selective small-molecule inhibitors of USP28 have been designed and reported 10, indicating that targeting USP28 may possess more translational significance. In cardiovascular disease, USP28 has recently been found to be involved in the regulation of diabetic cardiomyopathy by targeting peroxisome proliferator-activated receptor α (PPARα) 15, while the function of USP28 in cardiac hypertrophy is still unknown.

The purpose of this study was to clarify the regulatory role and mechanism of USP28 in cardiac hypertrophy. We showed that cardiomyocyte USP28 positively correlated with cardiac hypertrophy and USP28 deletion in cardiomyocytes significantly protected hearts from hypertrophy and dysfunction. Mechanistically, we identified tripartite motif-containing protein 21 (TRIM21) as the direct substrate of USP28. USP28 negatively regulates antioxidant response via deubiquitinating and stabilizing TRIM21. Our study illustrates a cardiomyocyte-specific USP28-TRIM21 axis in regulating hypertrophic cardiomyopathy and indicates USP28 as a pharmacological target for cardiac hypertrophy.

## Materials and Methods

### Animals

All *in vivo* experiments involving mouse and rat were approved by the Institutional Animal Ethics Committee of the 1st Affiliated Hospital of WMU (Approval document Number. WYYY-AEC-YS-2022-0073). Wildtype C57BL/6J mouse (WT), C57BL/6JGpt-Usp28^em1Cflox^/Gpt mouse (USP28^fl/fl^, strain No. T005113) and C57BL/6JGpt-H11^em1Cin(Myh6-iCre)^/Gpt mouse (*Myh6*-Cre, strain No. T004713) were obtained from GemPharmatech Co., Ltd (Nanjing, China). The cardiomyocyte-specific USP28 knockout mice (USP28CKO) was generated and maintained by crossing the USP28^fl/fl^ mice and the *Myh6*-Cre mice. Littermate USP28^fl/fl^ mice were used as the control counterpart of USP28CKO.

TAC model was established by a modified operation 16, 17: 6-8 weeks old male WT, USP28^fl/fl^ and USP28CKO mice were anesthetized with 2% isoflurane. After partial thoracotomy, blunt separation released the aortic arch. The aortic arch is then ligated with a 27G needle and 6-0 nylon suture. Sham operation was performed according to the same procedure except for aortic constriction. Mice were harvested 4 weeks after TAC or sham. For *in vivo* experiments involving USP28 inhibitor, selective USP28 inhibitor Otilonium Bromide 18 (10 mg/kg/day, dissolved in normal saline) was intragastric administrated at the end of 2^nd^ week after TAC. Mice were harvested 6 weeks after TAC (**Fig. 6A**).

Ang II model was established by osmotic mini- pump subcutaneous implantation: 6-8 weeks old male WT, USP28^fl/fl^ and USP28CKO mice were anesthetized with 2% isoflurane. Osmotic mini-pumps (1004, Alzet, Calif, USA) with Ang II (1000 ng/kg/min) or saline (same volume of Ang II) were subcutaneously implanted in the back of mouse for 4 weeks. An intelligent non-invasive blood pressure monitor (BP-2010A, Softron, Japan) was applied to measure the mice systolic blood pressure (SBP).

Cardiac function of mice was assessed by a Vevo 3100 high-resolution ultrasound imaging system. At the end of the study, all mice were euthanized under sodium pentobarbital anesthesia, and serum and heart tissues were collected for further analysis.

### Cell culture and transfection

HL-1 and NIH/3T3 were obtained from the Chinese Academy of Sciences' Type Culture Collection (Shanghai, China). Neonatal rat primary cardiomyocytes (NRPCs) were isolated from ventricle of neonatal Sprague-Dawley rats as described in our previous studies 19, 20. HL-1, NRPCs and NIH/3T3 were cultured in DMEM (Gibco, Germany) with 10% FBS (R223-00, Vazyme) as well as 1% streptomycin and penicillin.

*Usp28* and *Trim21* genes were silenced by small interfering RNA (siRNA; ACGGTTACCACAACTTAGA for si-rat-USP28, CUGGCAUUGUCUCCUUCUATT for si-rat-TRIM21, RIBOBIO, Guangzhou, China). Silencing of these genes were achieved by Lipo2000 (Thermo Fisher, 11668500).

Expression plasmids (Flag-USP28-WT (Mouse or Rat), Flag-USP28-C171A (Mouse), GFP-TRIM21 (Mouse) and HA-Ub/K48/K63 (Mouse)) were constructed by Genechem (Shanghai, China). Expression plasmid transfections were performed by Lipo3000 (Thermo Fisher, L3000150).

### Real-time (RT)-quantitative polymerase chain reaction (qPCR)

Total RNA of tissue and cell samples were extracted using TRIzol (15596018, Thermo Fisher). Isolated RNA was reverse transcribed to cDNA by a PrimeScript RT Kit (11201ES03, Yeasen). cDNA was processed for RT-qPCR using ChamQ Universal SYBR qPCR Master Mix (Q711-02, Vazyme). Primers were obtained from Sangon Biotech (**Table S1**).

### Statistical analysis

Data in this study are expressed as means± standard error (SEM). Student's t-test was used to identify difference between 2 groups. One-way analysis of variance (ANOVA) with multiple comparisons (Bonferroni's correction) was used to identify difference >2 groups. All statistical analysis was applied in GraphPad Pro Prism 8.0 (GraphPad, San Diego, CA). P value < 0.05 was accepted significant in this study.

An extended Materials and Methods section is available in the *Supplementary Information*.

## Results

### The expression of USP28 is increased in cardiac hypertrophy and is principally distributed in cardiomyocytes

**Figure 1:**
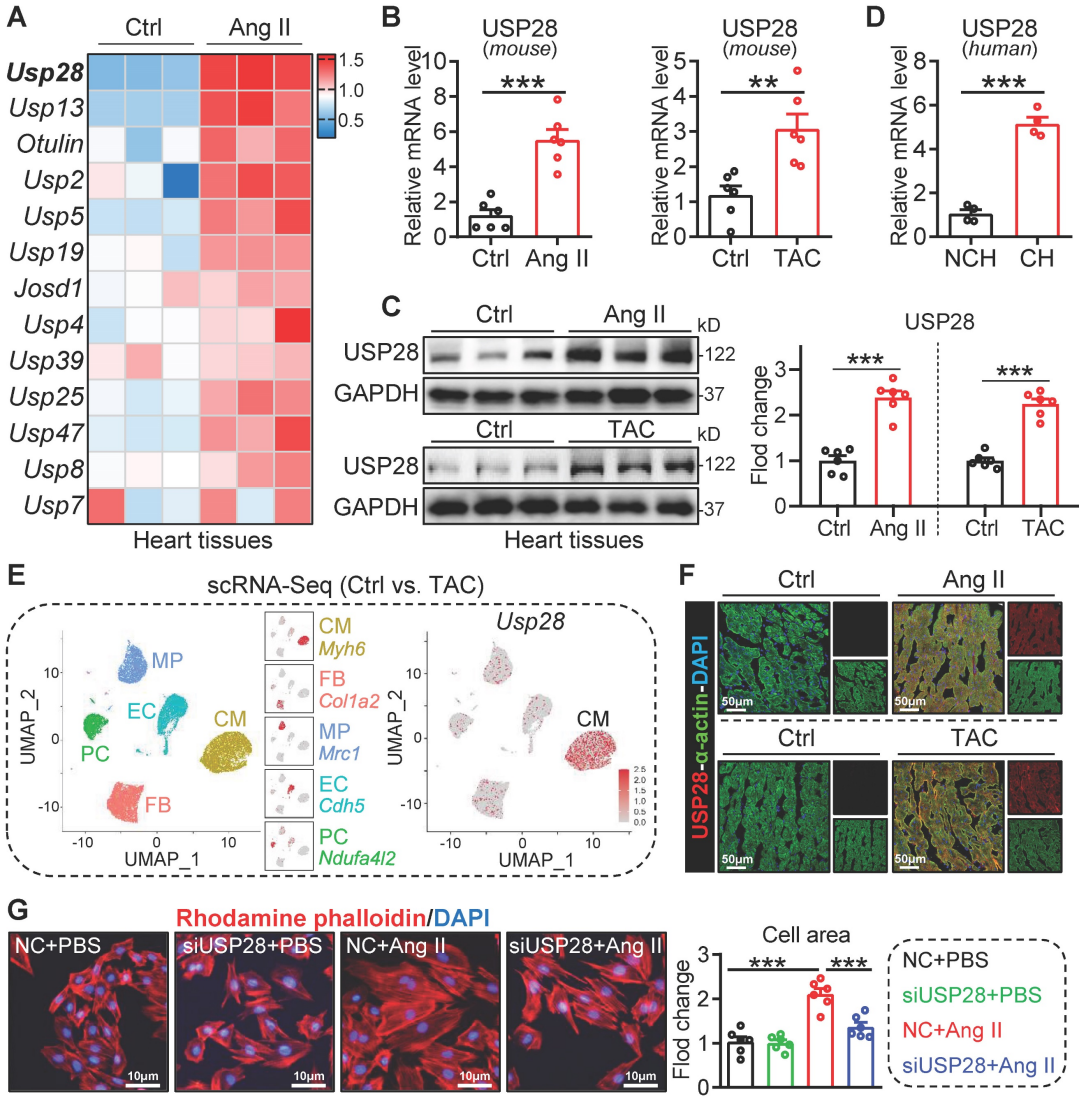
** The expression of USP28 is increased in cardiac hypertrophy and is principally distributed in cardiomyocytes. (A)** Heatmap showing the mRNA profile of DUB genes in Ang II-induced mouse hypertrophic hearts from our published transcriptome (n = 3; GSE221396). **(B)** The mRNA expression of *Usp28* in Ang II- or TAC- induced mouse cardiac hypertrophy (n = 6; ** P < 0.01, *** P < 0.001). **(C)** Western blot and quantitation of USP28 protein expression in mouse hypertrophic myocardium (n = 6; *** P < 0.001). **(D)** The *Usp28* mRNA level in human hypertrophic myocardium (NCH = non-cardiac hypertrophy, CH = cardiac hypertrophy; n = 4; *** P < 0.001). **(E)** scRNA-seq was performed in TAC mice hearts (For each group, single-cell suspensions from 3-4 hearts were pooled as 1 sample).** Left**, the UMAP dimensional reduction showing 5 main cell types of heart, including cardiomyocytes (CM), fibroblasts (FB), macrophages (MP), endothelial cells (EC) and pericytes (PC), and their specific marker genes. Approximate 17000 single heart cells (CM: 4484, EC: 3666, FB: 4320, MP: 2691, PC: 2154) were analyzed. **Right**, Biaxial scatter plot showing the expression pattern of *Usp28*. **(F)** Immunofluorescence staining of USP28 (red) and α-actin (green) in cardiac sections treated with Ang II (**upper**) or TAC (**below**). **(G)** NRPCs were transfected with siRNAs of NC (negative control) or USP28 followed by Ang II (1μM, 24h). TRITC-labeled rhodamine phalloidin staining (**left**) and the quantitative analysis (**right**) showed the surface area of NRPCs (n = 6; *** P < 0.001).

We first analyzed the mRNA profile of DUB genes in hearts of mice with or without Ang II infusion from our published transcriptome dataset (GSE221396) 21, in which we found that the up-regulation of* Usp28* was most prominent (**Fig. 1A**). Next, we validated that *Usp28* mRNA levels were increased in Ang II- or TAC- induced mouse hearts, when compared to the controls (**Fig. 1B**). The up-regulated protein level of USP28 was also confirmed in Ang II- or TAC- induced mouse hearts (**Fig. 1C**). Likewise, USP28 mRNA expression was increased in human hypertrophic myocardium (**Fig. 1D**). To examine the cellular localization of up-regulated USP28 in heart, we performed a scRNA-seq of approximate 17000 single heart cells from TAC-treated mice. Notably, the *Usp28* gene was principally distributed in cardiomyocytes (CM) (**Fig. 1E**). In line with scRNA-seq, immunofluorescence staining showed that the increased USP28 immunoreactivity was predominantly noted in α-actin^+^ cardiomyocytes (**Fig. 1F**). *In vitro*, USP28 protein expression was increased in Ang II-challenged cardiomyocyte (**Fig. S1A**). In addition, Ang II-induced expression of USP28 was mainly increased in cardiomyocytes, rather than non-cardiomyocytes (**Fig. S1B**). Functionally, we found that silencing USP28 suppressed Ang II or ISO-induced increase in surface area of NRPCs (**Fig. 1G** and **Fig. S1C-D**). Likewise, USP28 knockdown reduced the mRNA levels of hypertrophic genes (*Myh7*/*Nppa*) induced by Ang II *in vitro* (**Fig. S1E-F**). Together, these data indicated that cardiomyocyte USP28 expression is increased in cardiac hypertrophy and may mediate the hypertrophy of cardiomyocytes.

### Cardiomyocyte-specific USP28 knockout ameliorates TAC-induced myocardial dysfunction and hypertrophy

To investigate the effects of cardiomyocyte USP28 in cardiac hypertrophy, we generated cardiomyocyte-specific USP28 knockout mice (USP28CKO) via crossing USP28^fl/fl^ mice and *Myh6*-Cre mice (**Fig. S2A-B**). USP28CKO and littermate USP28^fl/fl^ (as the control counterpart) were subjected to TAC operation for 4 weeks. TAC model was confirmed by increased aortic blood flow velocity (**Fig. S3A**) and survival data (**Fig. S3B**).

**Figure 2:**
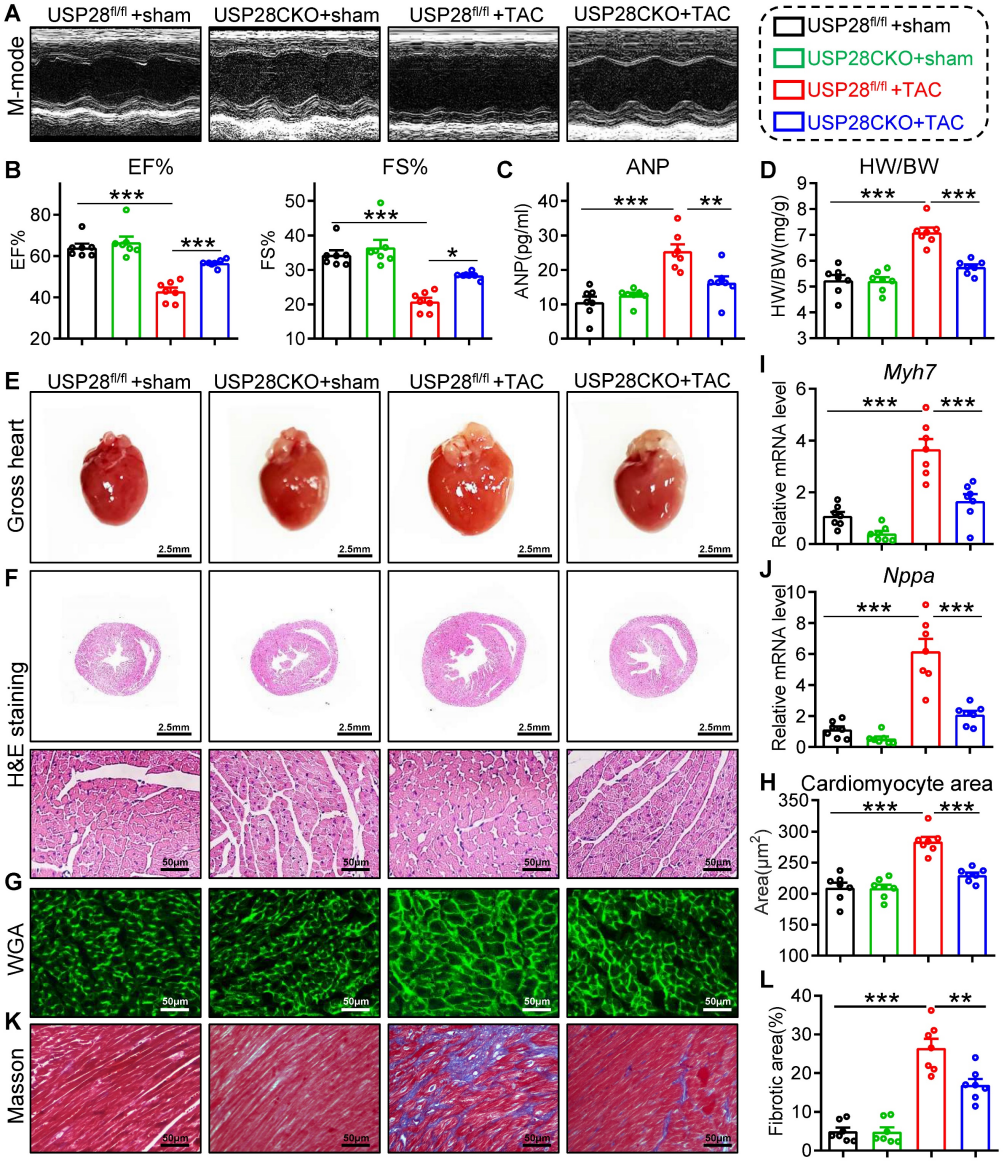
** Cardiomyocyte-specific USP28 knockout ameliorates TAC-induced myocardial dysfunction and hypertrophy.** USP28CKO and USP28^fl/fl^ mice were subjected to TAC or sham operations for 4 weeks. **(A)** M-mode echocardiographic images of left ventricle (LV) were assessed by non-invasive transthoracic echocardiography. **(B)** LV ejection fraction (EF) and fractional shortening (FS). **(C)** Serum ANP of indicated mice was tested by a mouse ANP ELISA Kit (F10062, Westang). **(D-E)** The ratio of heart weight (HW, mg) to body weight (BW, g) and representative gross-heart. **(F)** Representative H&E stained images of heart sections. **(G-H)** Representative WGA-stained sections and corresponding quantitative analysis of cardiomyocyte cross-sectional area. **(I-J)** The mRNA levels of *Myh7* and *Nppa* in heart tissues of indicated mice. **(K-L)** Representative Masson's trichome-stained sections and corresponding quantitative analysis of fibrotic area. (n = 7 for each group; ** P < 0.01, *** P < 0.001)

We first evaluated cardiac function by non-invasive echocardiography and found that TAC-treated USP28^fl/fl^ mice developed a significant cardiac dysfunction, while these dysfunctions were ameliorated in USP28CKO mice (**Fig. 2A-B and Table S2**). As shown in **Fig. 2C**, the increase of serum ANP induced by TAC was decreased in in USP28CKO mice. We measured cardiac morphology and discovered that USP28 knockout in cardiomyocytes markedly attenuated TAC-induced hypertrophic responses in whole heart, as indicated by the ratio of heart weight and body weight (HW/BW) (**Fig. 2D**) and the heart size (**Fig. 2E-F**). Examinations on cardiomyocyte cross-sectional area (**Fig. 2G-H**) and hypertrophic genes expression (*Myh7*/*Nppa***, Fig. 2I-J**) also showed that cardiomyocyte USP28 deletion reduced the TAC-induced cardiomyocyte hypertrophy in mice. Moreover, USP28 knockout in cardiomyocytes mitigated cardiac fibrosis, as assessed by Masson's trichome staining (**Fig. 2K-L and Fig. S3C**) and qPCR assay on mRNA levels of *Tgfb* and* Col1a1* (**Fig. S3D**). Together, cardiomyocyte-derived USP28 may be a crucial regulator of cardiac dysfunction and hypertrophy.

### Cardiomyocyte-specific USP28 deletion counteracts Ang II-induced hypertrophic heart failure

**Figure 3:**
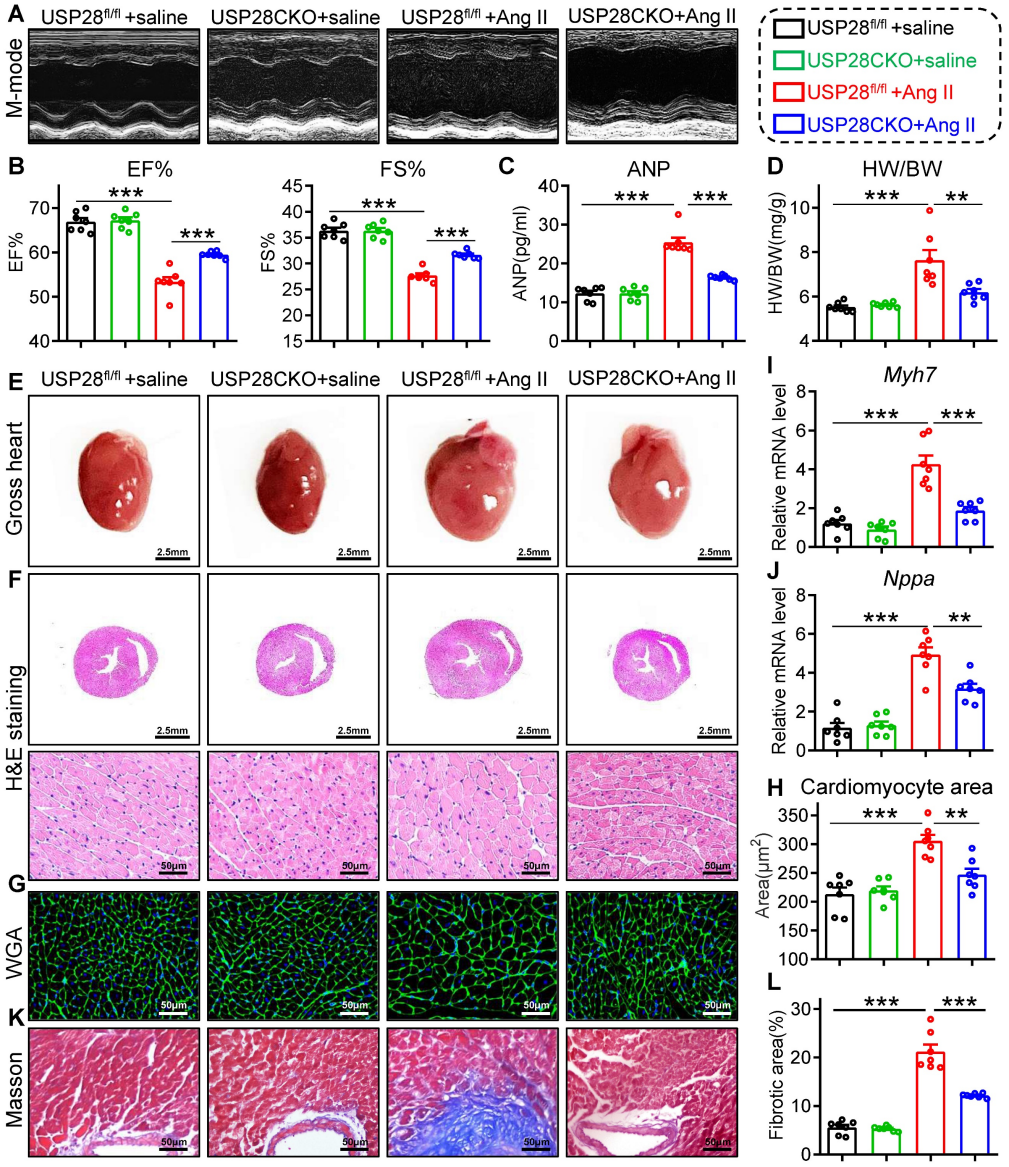
** Cardiomyocyte-specific USP28 deletion counteracts Ang II-induced hypertrophic heart failure.** USP28CKO and USP28^fl/fl^ mice were subcutaneously implanted with osmotic mini- pump (Saline or Ang II (1000 ng/kg/min)) for 4 weeks.** (A-B)** M-mode echocardiographic images, EF and FS of left ventricle. **(C)** Serum ANP of indicated mice. **(D-E)** The ratio of HW (mg) to BW (g) and representative gross-heart. **(F)** Representative H&E stained images of heart sections. **(G-H)** Representative WGA-stained sections and corresponding quantitative analysis of cardiomyocyte cross-sectional area.** (I-J)** The mRNA levels of *Myh7* and *Nppa* in heart tissues of indicated mice. **(K-L)** Representative Masson's trichome-stained sections and corresponding quantitative analysis of fibrotic area. (n = 7 for each group; ** P < 0.01, *** P < 0.001)

To further evaluate the role of cardiomyocyte-derived USP28 in hypertrophic heart failure, we generated another cardiac hypertrophy mouse model with Ang II infusion. USP28CKO and littermate USP28^fl/fl^ were subcutaneously implanted with osmotic mini-pump loaded Ang II for 4 weeks. Systolic blood pressure (SBP) did not differ in USP28CKO and USP28^fl/fl^ mice (**Fig. S4A**), indicating that cardiomyocyte USP28 does not affect blood pressure profile. In accordance with TAC model, non-invasive echocardiography revealed that Ang II-induced mice presented clear hallmarks of cardiac dysfunction, while USP28CKO mice exhibited higher EF% and FS% than USP28^fl/fl^ mice (**Fig. 3A-B and Table S3**). In addition, we showed USP28 knockout in cardiomyocytes reduced Ang II-induced increase in serum ANP (**Fig. 3C**). Ang II mice exhibited the hypertrophic response of whole heart as evidenced by HW/BW (**Fig. 3D**) and heart size (**Fig. 3E-F**), whereas cardiomyocyte-specific USP28 deletion reversed these changes. Consistently, USP28CKO decreased cardiomyocyte cross-sectional area (**Fig. 3G-H**) and mRNA levels of *Myh7* and *Nppa* in Ang II-challenged mice (**Fig. 3I-J**). Compared with USP28^fl/fl^ mice, USP28CKO showed a lower level of cardiac fibrosis under Ang II infusion (**Fig. 3K-L and Fig. S4B-C**). Overall, cardiomyocyte-specific USP28 knockout ameliorated Ang II-induced heart failure and hypertrophy.

### USP28 binds TRIM21 to regulate deubiquitinated modification and stability of TRIM21

**Figure 4:**
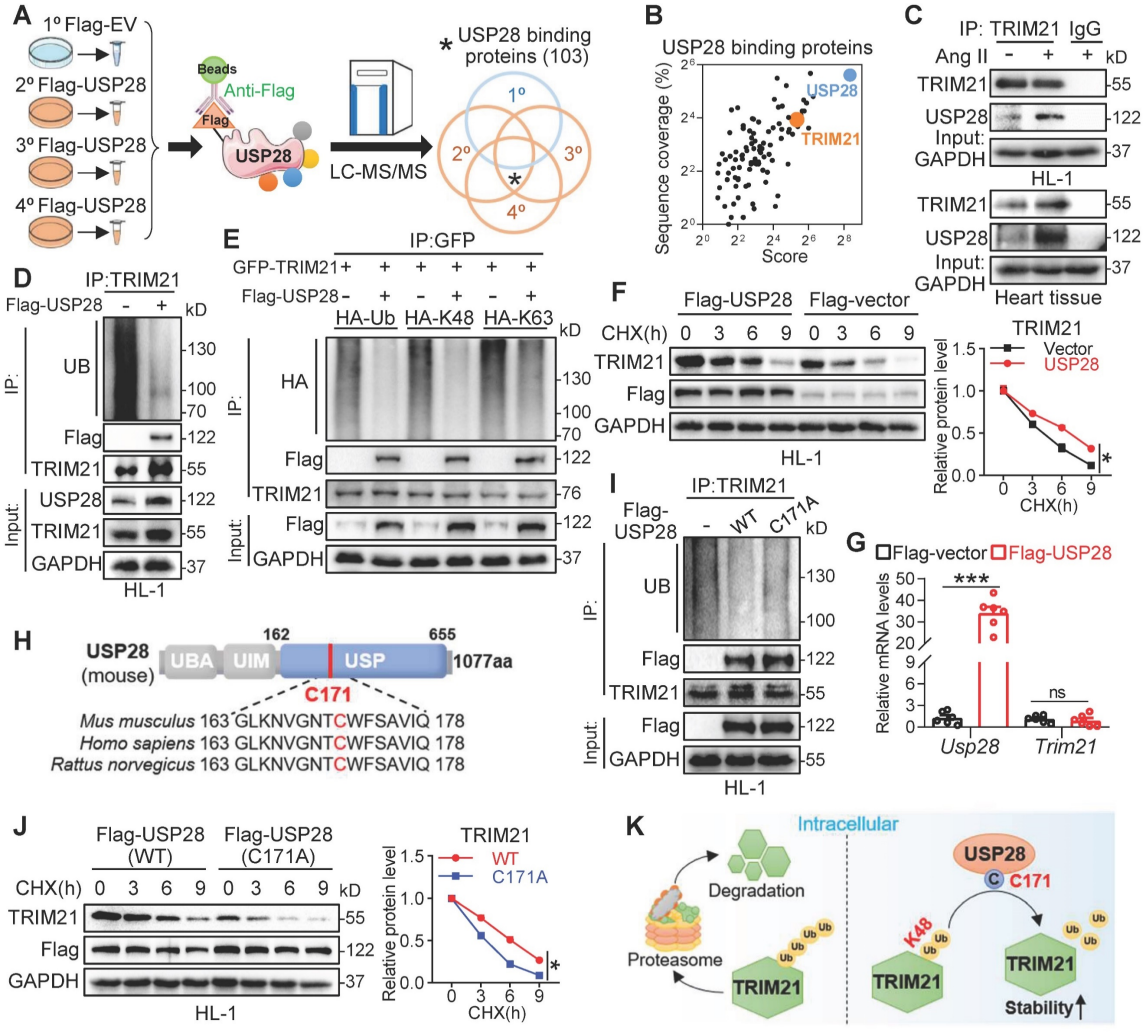
** USP28 binds TRIM21 to regulate deubiquitinated modification and stability of TRIM21. (A)** Schematic illustration of the co-IP combined LC-MS/MS for USP28 substrate screening. HL-1 was transfected with Flag-USP28 plasmids or Flag-vector, followed by Ang II stimulation (1μM, 24h). Anti-Flag and protein G-Sepharose beads were added to the cell samples for co-IP. The binding proteins were extracted, digested to peptide, and then subjected to LC-MS/MS analysis. **(B)** 2D plots with the USP28-IP score of the identified proteins on the x axis (revealing the enrichment in USP28-IP) and the Sequence coverage of proteins on the y axis (revealing the reliability of detected proteins). **(C)** Co-IP of endogenous USP28 and TRIM21 in lysates of HL-1 and heart tissues. **(D)** Flag-empty vector (EV) or USP28 were transfected into HL-1 and followed by 20μM MG132 for 6-8h. Ubiquitinated TRIM21 was enriched with anti-TRIM21 and then was detected with UB, Flag-USP28 and TRIM21. **(E)** GFP-TRIM21, HA-Ub, or its mutants reserving only K48 (HA-K48) or K63 (HA-K63) were transfected into NIH/3T3 with or without Flag-USP28 and then subjected to MG132. Co-IP was performed with anti-GFP and followed by western blot of HA, Flag-USP28 and TRIM21. **(F)** Protein levels of TRIM21 and Flag-USP28 in HL-1 expressing Flag-USP28 or Flag-vector with CHX (25μg/mL) pulse-chase stimulation and the quantitative analysis of TRIM21 (n = 3, * P < 0.05). **(G)** The mRNA levels of *Usp28* and *Trim21* in HL-1 expressing Flag-USP28 or Flag-vector (n = 6, *** P < 0.001, ns = no significant). **(H)** Schematic illustration of the domains and active site (C171) of USP28. **(I)** Flag-empty vector (EV) or USP28 (WT or C171A) were transfected into HL-1 and followed by MG132. Ubiquitinated TRIM21 was enriched with anti-TRIM21 and then was detected with UB, Flag-USP28 and TRIM21. **(J)** Protein levels of TRIM21 and Flag-USP28 in HL-1 expressing Flag-USP28 (WT or C171A) with CHX pulse-chase stimulation and the quantitative analysis of TRIM21 (n = 3, * P < 0.05). **(K)** Scheme for the mechanism of USP28 deubiquitinates TRIM21.

To identify the deubiquitinating substrates of USP28, we performed a co-IP combined LC-MS/MS analysis in Ang II stimulated HL-1 cells (**Fig. 4A**) and found 103 physically binding proteins of USP28 (**Fig. 4A-B**). Among the Top 20 candidate substrates (**Fig. S5A**), only one protein, TRIM21, has been reported to be directly involved in TAC-induced cardiac hypertrophy 22. Therefore, we speculated that USP28 may use TRIM21 as the crucial substrate in regulating cardiac hypertrophy. We then confirmed the endogenous interaction between USP28 and TRIM21 in Ang II-induced HL-1 and heart tissues using co-IP assay (**Fig. 4C**). Cellular co-localization analysis also showed that a large proportion of USP28 was colocalized with TRIM21 in Ang II-induced HL-1 cells (**Fig. S5B**). To examine the deubiquitinated modification of TRIM21 by USP28, we evaluated the ubiquitination level of TRIM21 in HL-1 cells expressing Flag-USP28 (USP28^OE^) and found that USP28^OE^ promoted the deubiquitination of TRIM21 (**Fig. 4D**). Further, we showed that USP28 reduced K48-linked ubiquitination of TRIM21 but not K63-linked ubiquitination (**Fig. 4E**). We applied a *de novo* protein synthesis inhibitor cycloheximide (CHX) to trace the stability of TRIM21 protein. USP28 increased the half-life of TRIM21 protein in HL-1 cells (**Fig. 4F**), whereas USP28 did not affect the mRNA transcript level of *Trim21* gene (**Fig. 4G**), indicating that USP28 increases TRIM21 level through preventing the degradation of TRIM21. Similarly, USP28 deletion in cardiomyocytes also reduced the protein level of TRIM21* in vivo* (**Fig. S5C-D**). As ubiquitinated proteins are degraded by the proteasome, we applied a proteasome inhibitor MG132 to demonstrate that USP28-dependent TRIM21 stability is proteasomal dependent. As expected, USP28 knockdown-induced TRIM21 degradation was reversed by MG132 (**Fig. S5E**). The conserved cysteine at 171 site (C171) is the catalytic motif in the deubiquitinating function of USP28 (**Fig. 4H**). We found that the enzyme-dead mutant of USP28 (C171A, mutation of cysteine to alanine at C171) failed to remove ubiquitin molecules from TRIM21 (**Fig. 4I**). Subsequently, the ability of USP28-C171A to maintaining the protein stability of TRIM21 was also decreased, compared with wildtype USP28 (**Fig. 4J**), although USP28-C171A still bound to TRIM21 (**Fig. S5F**). Taken together, USP28 binds to TRIM21 and regulates K48-linked deubiquitination and stability of TRIM21 via its active site C171 (**Fig. 4K**).

### USP28 negatively regulates antioxidant response in cardiomyocytes via its substrate TRIM21

**Figure 5:**
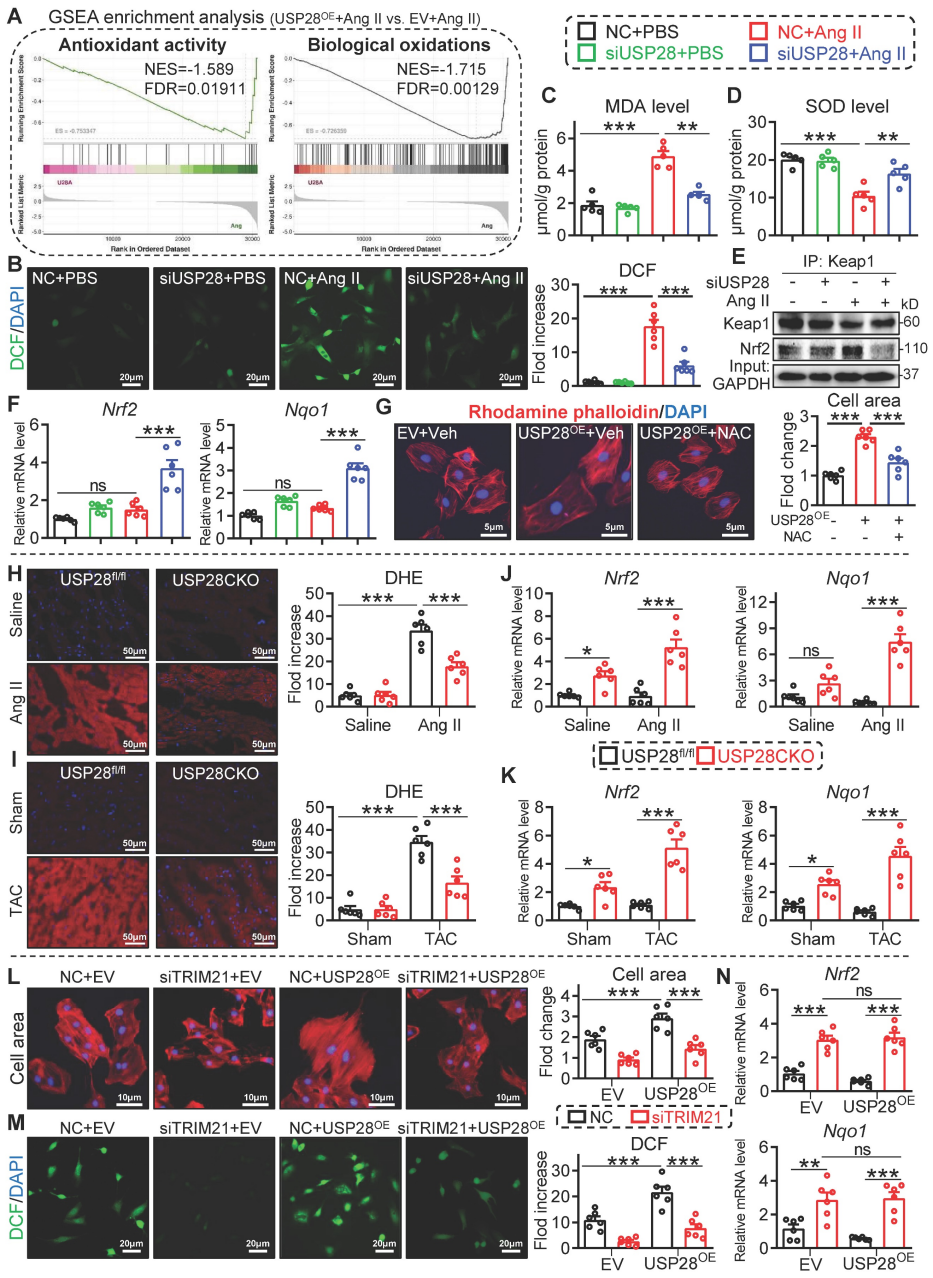
** USP28 negatively regulates antioxidant response via its substrate TRIM21. (A)** GSEA enrichment analysis of transcriptome in Ang II (1μM, 24h)-induced HL-1 cells expressing Flag-USP28 (USP28^OE^) or EV (NES: normalized enrichment score; FDR: false discovery rate). **B-F:** NRPCs were transfected with siRNAs of NC (negative control) or USP28 followed by Ang II (1μM, 24h) (n = 5 for C-D, n = 6 for B and F; ** P < 0.01, *** P < 0.001, ns = no significant).** (B)** DCF staining (**left**) and the quantitative analysis (**right**) showed the ROS production of NRPCs. **(C-D)** The levels of MDA and SOD. **(E)** Co-IP was performed with anti-Keap1 and followed by western blot of Keap1 and Nrf2.** (F)** The mRNA levels of *Nrf2* and *Nqo1*. **(G)** NRPCs were transfected with EV or USP28^OE^ for 24, and then treated with ROS scavenger NAC (1h; HY-B0215, MedChemExpress) following by the Ang II stimulation (1μM, 24h). TRITC-labeled rhodamine phalloidin staining (**left**) and the quantitative analysis (**right**) showed the surface area of NRPCs (n = 6; *** P < 0.001). **H-K:** USP28CKO and USP28^fl/fl^ mice were subjected to Ang II or TAC for 4 weeks (n = 6; * P < 0.05, ** P < 0.01, *** P < 0.001, ns = no significant). **(H-I)** Representative DHE staining and statistical analyses of ROS in myocardium of indicated mice. **(J-K)** The mRNA levels of *Nrf2* and *Nqo1* in heart tissues of indicated mice. **L-N:** NRPCs were transfected with siRNAs (NC or siTRIM21, 24h), and then transfected with EV or USP28^OE^ for 24 following by the Ang II stimulation (1μM, 24h) (n = 6; ** P < 0.01, *** P < 0.001, ns = no significant).** (L)** TRITC-labeled rhodamine phalloidin staining (**left**) and the quantitative analysis (**right**).** (M)** DCF staining (**left**) and the quantitative analysis (**right**). **(N)** The mRNA levels of *Nrf2* and *Nqo1*.

It has been reported that loss of TRIM21 protects heart from TAC-induced dysfunction and hypertrophy via activating nuclear factor erythroid 2-related factor 2 (Nrf2) antioxidant pathway 22. In this process, TRIM21 inhibits P62-regulated protein sequestration of Kelch-like ECH-associated protein-1 (Keap1) and maintains Keap1 levels to block Nrf2-antioxidant signaling in cardiomyocytes 22, 23. Here, we performed a transcriptome in Ang II-challenged HL-1 cells expressing Flag-USP28 (USP28^OE^) and showed that USP28^OE^ negatively regulates antioxidant activity and biological oxidations pathways (**Fig. 5A and Fig. S6A-B**). Therefore, the role of USP28-TRIM21 axis in Ang II-induced oxidative stress was examined. Silencing of USP28 led to a decline in the oxidative stress markers dichlorofluorescein (DCF) (**Fig. 5B**) and malondialdehyde (MDA) (**Fig. 5C**) and an increase in antioxidant enzyme superoxide dismutase (SOD) (**Fig. 5D**).

Additionally, silencing USP28 resulted in an activation in Nrf2 antioxidant pathway, as indicated by the dissociation of Nrf2 from Keap1 (**Fig. 5E**) and the increased expression of antioxidant *Nrf2* and *Nqo1* genes (**Fig. 5F**). These data indicate that USP28 positively regulates oxidative stress in cardiomyocytes. Using a ROS scavenger NAC, we showed that USP28^OE^-induced cardiomyocyte hypertrophy was attenuated by NAC (**Fig. 5G**), indicating the correlation between USP28-related cardiac hypertrophy and ROS production. Consistently, the regulation of USP28 deficiency on oxidative stress and antioxidant response was also examined in mouse heart tissues. Cardiomyocyte-specific USP28 knockout ameliorated Ang II or TAC-induced oxidative stress (**Fig. 5H-I and Fig. S6C-D**) and improved antioxidant response (**Fig. 5J-K**).

We then examined whether TRIM21 was required for the regulation of USP28 on antioxidant responses and cardiac hypertrophy. NRPCs were transfected with siRNA of TRIM21 (siTRIM21), and then transfected with USP28^OE^, followed by Ang II stimulation. As expected, USP28^OE^ aggravated Ang II-induced cardiomyocyte hypertrophy (**Fig. 5L**) and oxidative stress (**Fig. 5M**), while siTRIM21 prevented these exaggerated responses induced by USP28^OE^ (**Fig. 5L-M**). Notably, when TRIM21 was knocked down in cardiomyocytes, USP28^OE^ failed to inhibit the expression of antioxidant* Nrf2* and *Nqo1* (**Fig. 5N**). Taken together, these data indicated that USP28 negatively regulates Nrf2 antioxidant response to mediate cardiomyocyte injury via its substrate TRIM21.

### Selective USP28 inhibitor Otilonium Bromide (OB) abrogates TAC-induced hypertrophic heart failure and oxidative stress

**Figure 6:**
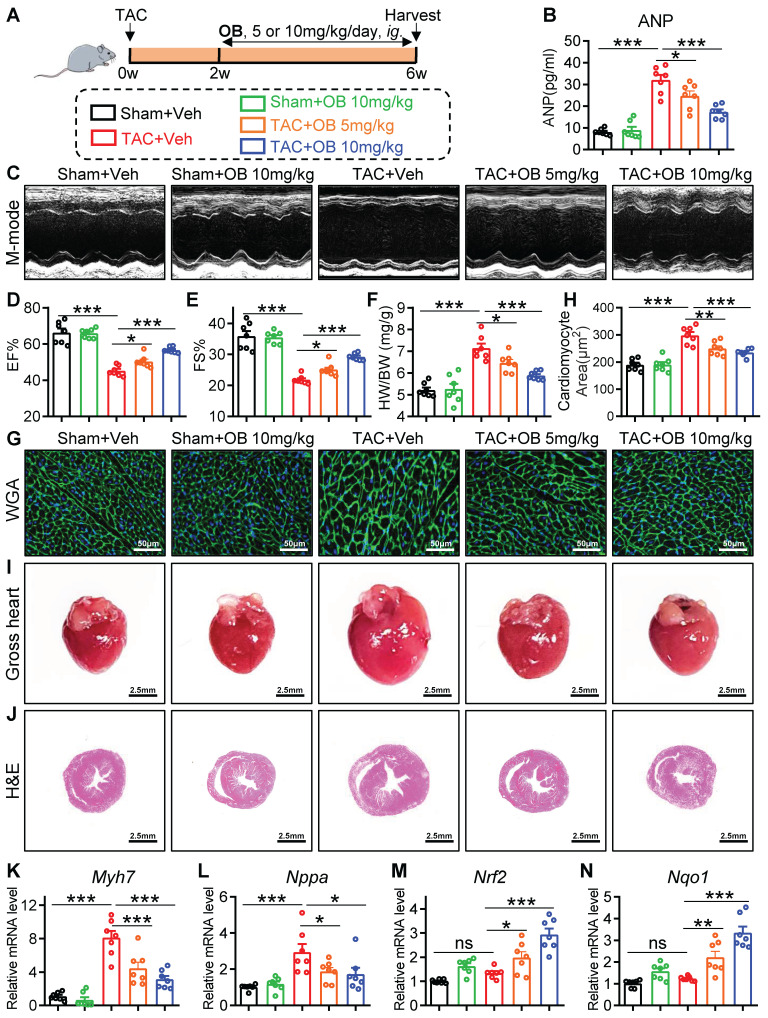
** Selective USP28 inhibitor Otilonium Bromide (OB) abrogates TAC-induced hypertrophic heart failure and oxidative stress. (A)** Mice of indicated group were underwent TAC for 6 weeks. Selective USP28 inhibitor OB (5 or 10 mg/kg/day) was intragastric administrated at the end of 2^nd^ week after TAC. All groups were harvested 6 weeks after TAC. **(B)** Serum ANP of indicated mice. **(C-E)** M-mode echocardiographic images, EF and FS of left ventricle. **(F)** The ratio of HW (mg) to BW (g). **(G-H)** Representative WGA-stained sections and corresponding quantitative analysis of cardiomyocyte cross-sectional area. **(I-J)** Representative gross-heart and H&E stained images of heart sections.** (K-L)** The mRNA levels of *Myh7* and *Nppa* in heart tissues of indicated mice. **(M-N)** The mRNA levels of *Nrf2* and *Nqo1*. (n = 7 for each group; ** P < 0.01, *** P < 0.001, ns = no significant)

Here, we examined whether specific inhibition of USP28 has a therapeutic effect on established cardiac hypertrophy induced by TAC. We applied a recently reported USP28 inhibitor Otilonium Bromide (OB, **Fig. S7A**), which showed a high selectivity of USP28 18. Wildtype (WT) mice were applied to TAC model for 6 weeks, and OB (10 mg/kg/day) was intragastric administrated at the end of 2^nd^ week after TAC (**Fig. 6A**). Firstly, echocardiography analysis confirmed the establishment of cardiac dysfunction 2 weeks after TAC (**Fig. S7B**). We then validated the inhibitory activity of OB against USP28 deubiquitinating TRIM21 (**Fig. S7C**) and maintaining TRIM21 protein stability (**Fig. S7D-E**). Next, we applied CHX to trace the time-course stability of TRIM21 under OB treatment. As shown in **Fig. S7F**, OB increased the degradation rate of TRIM21 protein in HL-1 cells. USP25 and USP28 are the most homologous proteins in USP family 10. We previously demonstrated that USP25 specifically deubiquitinates SERCA2a in hearts 21. To avoid the off-target effect of OB in cardiac hypertrophy, we measured the effect of OB on the USP25 deubiquitinating SERCA2a. As shown in **Fig. S7G**, OB did not inhibit the deubiquitination activity of USP25 to SERCA2a. These results showed that OB dose not inhibit USP25 enzyme activity and further confirmed the specificity of OB towards USP28 in cardiac hypertrophy.

As shown in **Fig. 6B**, OB suppressed TAC-induced increase in serum ANP in a dose-dependent manner. Non-invasive echocardiography revealed that TAC-induced cardiac dysfunction was dose-dependently attenuated by OB treatment (**Fig. 6C-E and Table S4**). Also, OB showed a dose- dependent inhibitory effect on the hypertrophic responses induced by TAC, as evidenced by HW/BW (**Fig. 6F**), WGA staining (**Fig. 6G-H**), heart size (**Fig. 6I**), H&E staining (**Fig. 6J**), and hypertrophic *Myh7* and *Nppa* gene expression (**Fig. 6K-L**). Likewise, mice treated with OB showed a lower level of cardiac fibrosis after TAC (**Fig. S7H-K**). The negative regulation of USP28 on antioxidant response was also examined in OB-treated mice. As shown in **Fig. 6M-N,** OB administration significantly increased antioxidant *Nrf2/Nqo1* gene expression in a dose-dependent manner. In addition, we added a group of mice with OB alone to examine the drug safety. As shown in **Fig. 6B-L** and **Fig. S7H-K**, administration with OB alone at the present dosage (10mg/kg) did not cause cardiac dysfunction, hypertrophic responses, and interstitial fibrosis. Overall, selective USP28 inhibitor OB abrogates TAC-induced established hypertrophic heart failure and oxidative stress in mice.

## Discussion

DUBs are closely involved in the pathophysiological regulations of cardiac hypertrophy 8, and our research group also found that two DUBs (USP25 and JOSD2) can inhibit cardiac hypertrophy and dysfunction by stabilizing SERCA2a and thereby maintaining calcium handling in cardiomyocytes 21, 24. Therefore, DUBs family can serve as a potential bank for seeking new therapeutic targets for HF. In this study, we found the up-regulation of USP28 in cardiac hypertrophy and its cellular localization of cardiomyocytes. The structure and amino acid sequence of USP28 and USP25 are highly homologous 10, while USP28 is preferentially expressed in myocardial tissue 9. Knockout or inhibition of USP28 can effectively mitigate various cancers 12, immune system diseases 13 and neurodegenerative diseases 11. In this study, USP28CKO reduced TAC- or Ang II-induced cardiac dysfunction and hypertrophy, suggesting the potential of USP28 as a therapeutic target for cardiac hypertrophy.

DUBs possess their abilities mainly through specific substrate proteins 7. Here, we performed co-IP/MS analysis and identified TRIM21 as a potential substrate of USP28. We then have investigated the binding of USP28 and TRIM21 and the maintenance of TRIM21 stabilization by USP28 via K48-linked deubiquitination. TRIM21 itself is an E3 ubiquitin ligase 25, and its role and mechanism in heart disease have been extensively reported 22, 23, 26, 27.

Negative regulation of Nrf2-antioxidant axis is a common mechanism in TRIM21-mediated heart diseases, and has been verified in TAC-induced cardiac hypertrophy 22, doxorubicin-induced cardiotoxicity 23, and myocardial infarction-induced atrial remodeling 26. In this process, TRIM21 ubiquitylates and degrades P62, thereby inhibiting P62-regulated protein sequestration of Keap1 and maintaining Keap1 levels to block Nrf2-antioxidant signaling in cardiomyocytes 22, 23. Thus, we consider that USP28, which increases TRIM21 stability in cardiomyocytes, down-regulates Nrf2-antioxidant response by the known TRIM21-P62-Keap1 axis. Here, we detected the interaction between TRIM21 and P62 and showed that siUSP28 reduced the interaction between TRIM21 and P62, indicating that USP28 further affects the TRIM21-P62 axis (**Fig. S6E**). However, it has been reported that TRIM21 is widespread expressed and functions in the pathophysiology of multi-organs 28. Compared with the direct intervention of TRIM21, targeting USP28 has significant cardiomyocyte specificity and may avoid potential side-effects 9. On the other hand, compared with the rapid development of USP28 inhibitors 10, the TRIM21 specific inhibitor has not been reported, suggesting that targeting the protein structure of TRIM21 might be difficult to design small-molecule drugs. Therefore, we present that USP28 may be a more cardiomyocyte-specific and druggable therapeutic target than TRIM21 for cardiac hypertrophy.

In this study, we showed that USP28 negatively regulates antioxidant activity pathway through deubiquitinating TRIM21 in cardiomyocytes. Cardiac hypertrophy promotes the ROS generation via NADPH oxidases, mitochondrial dysfunction, and xanthine oxidase, and the oxidative stress in cardiomyocyte further induces structural and functional injuries 29. ROS can be cleared by Nrf2 antioxidant signaling pathway 29. Increasing the expression or activation of Nrf2 has been shown to protect against cardiac remodeling and dysfunction in experimental models 29. Here, we have investigated the negative regulation of antioxidant response by USP28 both* in vitro* and *in vivo*. Through deubiquitinating TRIM21, USP28 inhibits Nrf2 expression to increase cellular ROS level, which promotes cardiomyocyte hypertrophy. At present, the role and mechanism of USP28 in regulating oxidative stress have not been reported, and our results provide a theoretical basis for the study of USP28-oxidative stress axis in other diseases.

In recent years, several inhibitors targeting USP28 have been designed and discovered 10. However, given that USP28 and USP25 are the most homologous proteins in USP family, most inhibitors target both with low selectivity 10. Interestingly, in many cases, USP28 and USP25 play opposite roles in the same disease, such as cardiac hypertrophy, where USP25 is a protective protein 21 but USP28 promotes this disease development. Therefore, it is necessary to use highly efficient and specific inhibitor against USP28 in the treatment of myocardial hypertrophy. Here, we applied a recently reported USP28-specific inhibitor OB and showed that selective inhibition of USP28 by OB abrogates TAC-induced established hypertrophic heart failure and oxidative stress, indicating OB as a potential candidate for the treatment of heart failure.

Apart from the USP28-TRIM21 interaction, USP28 has been reported to deubiquitinate some other substrate proteins 12, such as Cyclin E1 30 and NICD1 31. Although these substrates have a low association with cardiac hypertrophy and were not present in our LC-MS/MS, we cannot completely rule out that USP28 regulates cardiac hypertrophy through these proteins. Our study shows that USP28 is a detrimental DUB in cardiac hypertrophy, however, a recent study showed that USP28 protects mouse heart against diabetic cardiomyopathy by targeting PPARα 15. Also, we did not find the potential PPARα-USP28 interaction in TAC-induced mouse heart from our LC-MS/MS dataset. These results suggest that the function and substrate of cardiomyocyte USP28 are different in various pathological states of the hearts. Therefore, the clinical application of USP28 inhibitors should be cautious and depend on the diagnosis of specific heart diseases. For small-molecule USP28 inhibitor, although OB is the most specific inhibitor against USP28 published so far, it still can target USP25 at a low affinity level, and we cannot rule out its specificity for non-DUBs targets. Considering the functional differences among DUBs, highly selective USP28 inhibitors is worthy of further design and exploration.

Collectively, we showed that cardiomyocyte USP28 deubiquitinates and stabilizes TRIM21 to negatively regulate antioxidant response, increasing oxidative stress in cardiomyocytes and promoting cardiac hypertrophy and dysfunction. We also demonstrated that either cardiomyocyte-specific knockout or pharmacologically inhibition of USP28 significantly protected hearts against pathological cardiac hypertrophy in mice. Given TRIM21 is not feasible as a therapeutic target for cardiac hypertrophy, due to its widespread expression in multi-organs, the cardiomyocyte-specific USP28-TRIM21 axis may avoid the potential and systemic side-effects induced by targeting TRIM21 alone, suggesting that targeting USP28 is a more specific strategy for cardiac hypertrophy therapy.

## Supplementary Material

Supplementary materials and methods, figures and tables.
