# Supplementary material for: Cardiomyocyte-derived USP28 negatively regulates antioxidant response and promotes cardiac hypertrophy via deubiquitinating TRIM21
Source: Theranostics. 2024 Sep 30;14(16):6236–48. doi: 10.7150/thno.99340 (PMC11488095; doi:10.7150/thno.99340)
Supplement: Supplementary file 1 — Supplementary materials and methods, figures and tables. [file thnov14p6236s1.pdf]

Cardiomyocyte-derived USP28 negatively regulates antioxidant response and promotes cardiac hypertrophy via deubiquitinating TRIM21

Supplementary Information

Supplementary Information Contents:

1. Extended Materials and Methods
2. Supplementary Figure S1-7
3. Supplementary Table S1-4

Extended Materials and Methods

Reagents

Ang II (HY-13948) and OB (HY-B0499A) were obtained from MedChemExpress (New Jersey, USA). The solubility of Ang II for *in vivo* experiment was improved by adding cosolvents (DMSO, PEG300 and Tween-80) according to the Product Data Sheet. MG132 (T2154) was purchased from TargetMol (shanghai, China). Antibodies against TRIM21 (12108-1-AP, 1:1000), Flag (20543-1-AP, 1:1000), GFP (66002-1-Ig, 1:1000), HA (81290-1-RR, 1:1000), Nrf2 (16396-1-AP, 1:1000), Keap1 (10503-2-AP, 1:1000) and GAPDH (60004-1-IG, 1:1000) were purchased from Proteintech (Wuhan, China). Antibody against UB (3936, 1:1000) was purchased from CST (Danvers, MA, USA). Antibodies against USP28 (ab188240, 1:1000 for western blot and 1:200 for immunofluorescence-double staining) and α-actin (ab9465, 1:200 for immunofluorescence-double staining) were purchased from Abcam.

Animals

All *in vivo* experiments involving mouse and rat were approved by the Institutional Animal Ethics Committee of the 1st Affiliated Hospital of WMU (Approval document Number. WYYY-AEC-YS-2022-0073) and strictly followed the NIH Guide for the Care and Use of Laboratory Animals. Wildtype C57BL/6J mouse (WT), C57BL/6JGpt-Usp28^{em1Cflox}/Gpt mouse (USP28$^{fl/fl}$, strain No. T005113) and C57BL/6JGpt-H11$^{em1Cin(Myh6\text{-}iCre)}$/Gpt mouse (*Myh6*-Cre, strain No. T004713) were obtained from GemPharmatech Co., Ltd (Nanjing, China). The cardiomyocyte-specific USP28 knockout mice (USP28CKO) was generated and maintained by crossing the USP28$^{fl/fl}$ mice and the *Myh6*-Cre mice. Littermate USP28$^{fl/fl}$ mice were used as the control counterpart of USP28CKO.

TAC model was established by a modified operation [1, 2]: 6-8 weeks old male WT, USP28$^{fl/fl}$ and USP28CKO mice were anesthetized with 2% isoflurane. After partial thoracotomy, blunt separation released the aortic arch. The aortic arch is then ligated with a 27G needle and 6-0 nylon suture. Sham operation was performed according to the same procedure except for aortic constriction. Mice were harvested 4 weeks after TAC or sham. For *in vivo* experiments involving USP28 inhibitor, selective USP28 inhibitor OB [3] (10 mg/kg/day, dissolved in normal saline) was intragastric administrated at the end of 2nd week after TAC. Mice were harvested 6 weeks after TAC (**Fig. 6A**).

Ang II model was established by osmotic mini- pump subcutaneous implantation: 6-8 weeks old male WT, USP28$^{fl/fl}$ and USP28CKO mice were anesthetized with 2% isoflurane. Osmotic mini-pumps (1004, Alzet, Calif, USA) with Ang II (1000 ng/kg/min) or saline (same volume of Ang II) were subcutaneously implanted in the back of mouse for 4 weeks. An intelligent non-invasive blood pressure monitor (BP-2010A, Softron, Japan) was applied to measure the mice systolic blood pressure (SBP).

Cardiac function of mice was assessed by a Vevo 3100 high-resolution ultrasound imaging system. At the end of the study, all mice were euthanized under sodium pentobarbital anesthesia,

and serum and heart tissues were collected for further analysis.

scRNA-seq

Hearts were obtained from TAC-treated mice and dissociated into single cells by dissociation solution. For each group, single-cell suspensions from 3-4 hearts were pooled as 1 sample. Flow cytometry is used to sort cells, selecting viable single cells with a cell survival rate of ≥ 95%. Cells with low quality (<200 or >5000 genes per cell and >25% mitochondrial genes in the cell) were filtered out. Single-cell suspensions were loaded onto 10x Chromium to capture single cells by 10X Genomics Chromium Single-Cell 3' kit. The cDNA amplification, library construction and sequencing were performed by LC-BIO technologies Co., Ltd (Hangzhou, China). CellRangerv6.0 (https://github.com/10XGenomics/cellranger) analyse the off-machine single-cell data (fastq format) and compares it with the Mus musculus GRCm38.96 reference genome. We have performed principal components analysis (PCA) before dimensionality reduction. Seurat clustering was applied for identifying cell clusters. UMAP clustering algorithm was used to cluster the cells for dimensionality reduction. We used marker genes for manual identification of cell populations.

Histopathological evaluation

The cardiomyocyte localization of USP28 in cardiac tissues was analyzed by immunofluorescence-double staining. OCT-embedded slices were co-incubated with α-actin antibody and USP28 antibody, followed by incubation with Alexa Fluor488-labeled secondary antibody (1:1000, Abcam) or TRITC-labeled secondary antibody (1:1000, Abcam) for 1 h. DAPI was applied to stain the nuclei. 5 μm thick paraffin-embedded slices were stained with hematoxylin and eosin (H&E) (G1120, Solarbio, Beijing, China) or Masson's trichrome (G1340, Solarbio, Beijing, China), the former for cardiac histologic analysis and the latter for collagen fiber analysis. 5 μm thick OCT-embedded frozen sections were stained with wheat germ agglutinin (WGA, GTX01502, Gene Tex) for cardiomyocyte area assessment. 5 μm thick OCT-embedded frozen sections were incubated with 10 μM dihydroethidium (DHE) (Yeasen, Shanghai, China) for ROS assessment. Above images were taken by optical or fluorescence microscope (80i, Nikon, Japan). slices

Cell culture and transfection

HL-1 and NIH/3T3 were obtained from the Chinese Academy of Sciences' Type Culture Collection (Shanghai, China). Neonatal rat primary cardiomyocytes (NRPCs) were isolated from ventricle of neonatal Sprague-Dawley rats as described in our previous studies [4, 5]. HL-1, NRPCs and NIH/3T3 were cultured in DMEM (Gibco, Germany) with 10% FBS (R223-00, Vazyme) as well as 1% streptomycin and penicillin.

Usp28 and *Trim21* genes were silenced by small interfering RNA (siRNA; ACGGTTACCACAACTTAGA for si-rat-USP28, CUGGCAUUGUCUCCUUCUATT for si-rat-

TRIM21, RIBOBIO, Guangzhou, China). Silencing of these genes were achieved by Lipo2000 (Thermo Fisher, 11668500).

Expression plasmids (Flag-USP28-WT (Mouse or Rat), Flag-USP28-C171A (Mouse), GFP-TRIM21 (Mouse) and HA-Ub/K48/K63 (Mouse)) were constructed by Genechem (Shanghai, China). Expression plasmid transfections were performed by Lipo3000 (Thermo Fisher, L3000150).

Co-IP combined LC-MS/MS analysis

HL-1 were transfected with Flag-USP28 plasmids or Flag-vector, followed by Ang II stimulation. Anti-Flag and protein G-Sepharose beads were added to the cell samples for co-IP. The binding proteins were extracted from co-IP beads using SDT lysis buffer. Protein was digested to peptide via FASP method. The LC-MS/MS was then carried out by BIOPROFILE (Shanghai, China). The MS data were analyzed using MaxQuant software version 2.0.1.0. MS data were searched against the UniProtKB Mus musculus database (55286 total entries, downloaded 01/04/2023). The trypsin was seleted as digestion enzyme. The maximal two missed cleavage sites and the mass tolerance of 4.5 ppm for precursor ions and 20 ppm for fragment ions were defined for database search. Carbamidomethylation of cysteines was defined as fixed modification, while acetylation of protein N-terminal, oxidation of Methionine were set as variable modifications for database searching. The database search results were filtered and exported with <0.01% false discovery rate (FDR) at peptide-spectrum-matched level, and protein level, respectively. The candidate substrate proteins were screened out according to the score of detected proteins.

Transcriptome

One transcriptome was carried out in hearts of mice with or without Ang II infusion, and the data presented in the study are deposited in the GEO repository (GSE221396). Another transcriptome was carried out in Ang II-induced HL-1 cells expressing Flag-USP28 or EV. TRIzol reagent (Thermo Fisher) was used to extract total RNA from heart tissues or cells. Library construction, sequencing and GSEA enrichment analysis were performed by LC-Bio technologies Co., Ltd (Hangzhou, China). FASTQ sequence data were mapped to Mus musculus GRCm38.96 reference genome. The reads per gene were counted by using "HTseq". Gene expression was presented as FPKM. The differentially expressed genes (DEGs) were defined by fold change > 1.5 or < 0.67, and $P < 0.05$.

Reactive oxygen species (ROS) determination

The amount of malondialdehyde (MDA) and superoxide dismutase (SOD) was measured by assay kits (S0131S for MDA and S0101S for SOD, Beyotime, Shanghai, China) according to the product datasheets. For cell samples, we use the number of cells to standardize. For tissue samples, we use the amount of protein to standardize.

Cardiomyocyte surface area and DCF staining *in vitro*

TRITC-labeled rhodamine phalloidin staining (CA1610, Solarbio, Beijing, China) was applied to measure the surface area of cardiomyocytes. 2′,7′-dichlorofluorescin diacetate (DCF; 4091-99-0, Solarbio) staining was performed to determine the ROS in cardiomyocytes. DAPI was applied to stain the nuclei and images were taken by fluorescence microscope (TI-S, Nikon, Japan).

Western blot and co-IP

Proteins were extracted from cell and tissue samples by RIPA (P0013B, Beyotime). Equal amounts of proteins were loaded on SDS-PAGE gels and then transferred to PVDF membranes. After blocking with BSA, membranes were incubated with primary antibodies (overnight, 4°C), followed by corresponding HPR-labeled secondary antibodies. Chemiluminescence measurement was performed by ECL fluorescence detection kit (P10300, NCM). For co-IP, the tissue or cell lysates were incubated with primary antibodies (overnight, 4°C). Proteins was then precipitated with protein G-Sepharose beads (6-12h, 4°C). After washed by PBS, protein G-Sepharose beads were prepared for western blot.

Human samples

Human myocardial samples were collected as previously described [6]. Written informed consent was obtained and the experiments involving human samples were approved by the Ethics Committee of the 1st Affiliated Hospital of WMU (Approval document Number. KY2022-156).

Real-time (RT)-quantitative polymerase chain reaction (qPCR)

Total RNA of tissue and cell samples were extracted using TRIzol (15596018, Thermo Fisher). Isolated RNA was reverse transcribed to cDNA by a PrimeScript RT Kit (11201ES03, Yeasen). cDNA was processed for RT-qPCR using ChamQ Universal SYBR qPCR Master Mix (Q711-02, Vazyme). Primers were obtained from Sangon Biotech (**Table S1)**.

Statistical analysis

Data in this study are expressed as means± standard error (SEM). Student's t-test was used to identify difference between 2 groups. One-way analysis of variance (ANOVA) with multiple comparisons (Bonferroni's correction) was used to identify difference >2 groups. All statistical analysis was applied in GraphPad Pro Prism 8.0 (GraphPad, San Diego, CA). P value <0.05 was accepted significant in this study.

Reference

1. Richards DA, Aronovitz MJ, Calamaras TD, Tam K, Martin GL, Liu P, et al. Distinct Phenotypes Induced by Three Degrees of Transverse Aortic Constriction in Mice. Sci Rep. 2019; 9: 5844.

2. Liu B, Li A, Gao M, Qin Y, Gong G. Modified Protocol for A Mouse Heart Failure Model Using Minimally Invasive Transverse Aortic Constriction. STAR Protoc. 2020; 1: 100186.

3. Xu Z, Wang H, Meng Q, Ding Y, Zhu M, Zhou H, et al. Otilonium Bromide acts as a selective USP28 inhibitor and exhibits cytotoxic activity against multiple human cancer cell lines. Biochem Pharmacol. 2023; 215: 115746.
4. Han J, Dai S, Zhong L, Shi X, Fan X, Zhong X, et al. GSDMD (Gasdermin D) Mediates Pathological Cardiac Hypertrophy and Generates a Feed-Forward Amplification Cascade via Mitochondria-STING (Stimulator of Interferon Genes) Axis. Hypertension. 2022; 79: 2505-18.
5. Ye B, Shi X, Xu J, Dai S, Xu J, Fan X, et al. Gasdermin D mediates doxorubicin-induced cardiomyocyte pyroptosis and cardiotoxicity via directly binding to doxorubicin and changes in mitochondrial damage. Transl Res. 2022; 248: 36-50.
6. Han J, Fang Z, Han B, Ye B, Lin W, Jiang Y, et al. Deubiquitinase JOSD2 improves calcium handling and attenuates cardiac hypertrophy and dysfunction by stabilizing SERCA2a in cardiomyocytes. Nat Cardiovasc Res. 2023; 2: 764-77.

Supplementary Figures:

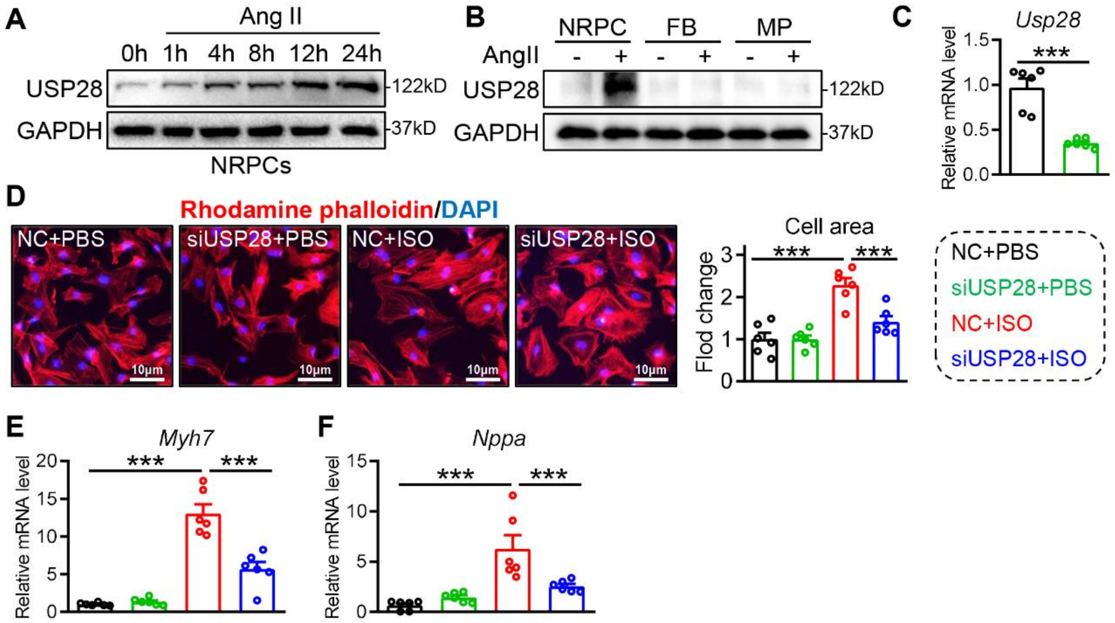

Figure S1 (A) Representative immunoblot for USP28 in 1μM Ang II-induced NRPCs. **(B)** Protein expression of USP28 in NRPCs, fibroblasts (FB) and bone marrow derived macrophage (MP) stimulated by Ang II (1 μM, 24h). **C-F:** NRPCs were transfected with siRNAs of NC (negative control) or USP28 followed by ISO (20 μM, 24h) or Ang II (1 μM, 24h). **(C)** The mRNA levels of *Usp28*. **(D)** TRITC-labeled rhodamine phalloidin staining (**left**) and the quantitative analysis (**right**) showed the surface area of NRPCs. **(E-F)** The mRNA levels of *Myh7* and *Nppa*. (n=6; *** P<0.001).

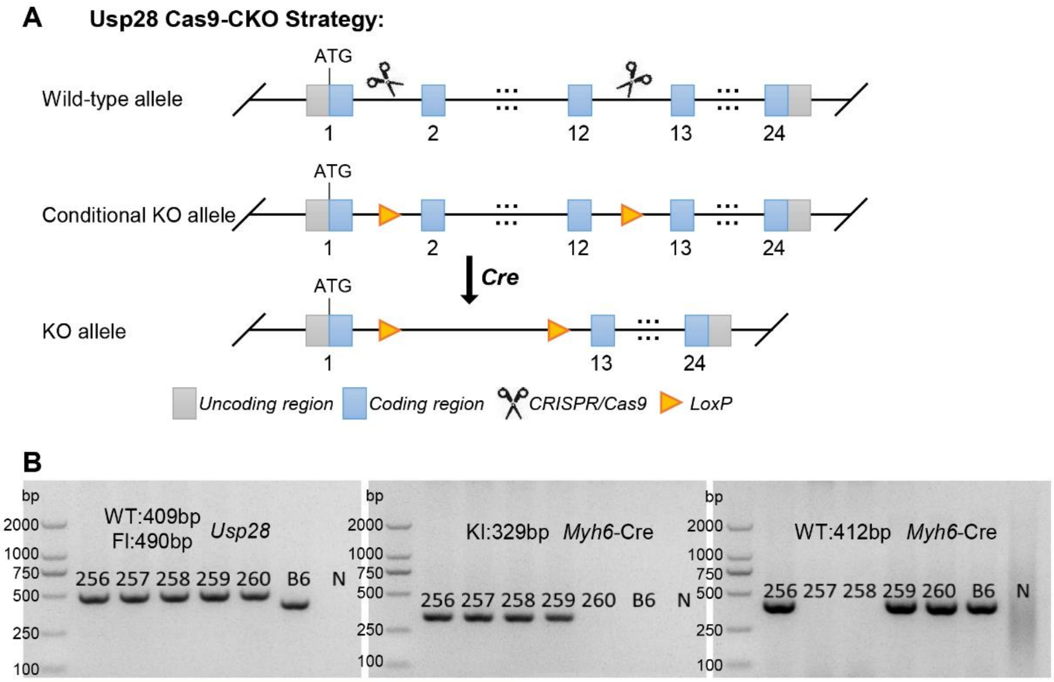

Sample Name	#*Usp28*#	#*Myh6*-Cre#
256	fl/fl	ki/wt
257	fl/fl	ki/ki
258	fl/fl	ki/ki
259	fl/fl	ki/wt
260	fl/fl	wt/wt

B6: Negative Control

N: No-Template Control

Figure S2 (A) The schematic diagram of CRISPR/Cas9 technology to edit the *Usp28* gene: The *Usp28* gene has 10 transcripts, exon2-exon12 of *Usp28-201* (ENSMUST00000047349.7) transcript is recommended as the knockout region. The region contains 1229bp coding sequence. The flox mice will be knocked out after mating with mice expressing Cre recombinase, resulting in the loss of function of the target gene in specific tissues and cell types. **(B)** The primers of *Usp28* (WT:409bp, FI:490bp) and *Myh6-Cre* (WT:412bp, KI:329bp) were respectively used for PCR to identify the genotype of mice. (P: Positive control; B6: Negative control; N: No-template control).

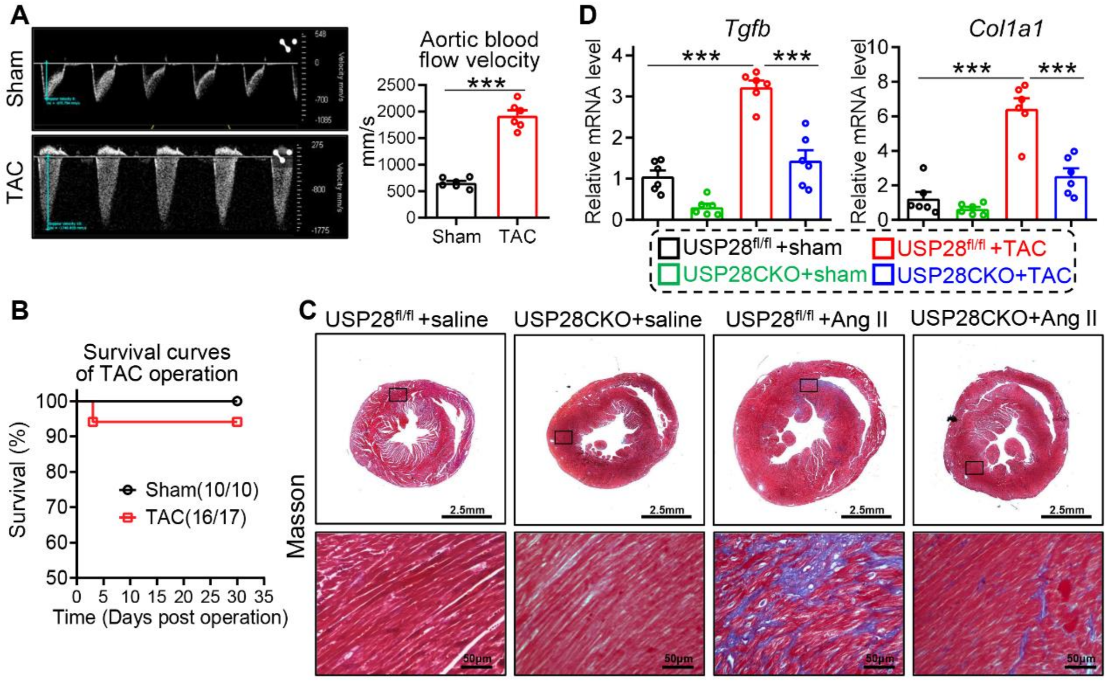

Figure S3 USP28CKO and USP28$^{fl/fl}$ mice were subjected to TAC or sham operations for 4 weeks. **(A)** Flow doppler ultrasound images of the aortic arch of indicated mice and the quantitative analysis of transverse aortic flow velocity (n=6). **(B)** Kaplan-Meier survival curves. **(C)** Masson's trichome-stained sections. **(D)** The mRNA levels of *Tgfb* and *Col1a1* in heart tissues of indicated mice (n=7). (*** P<0.001)

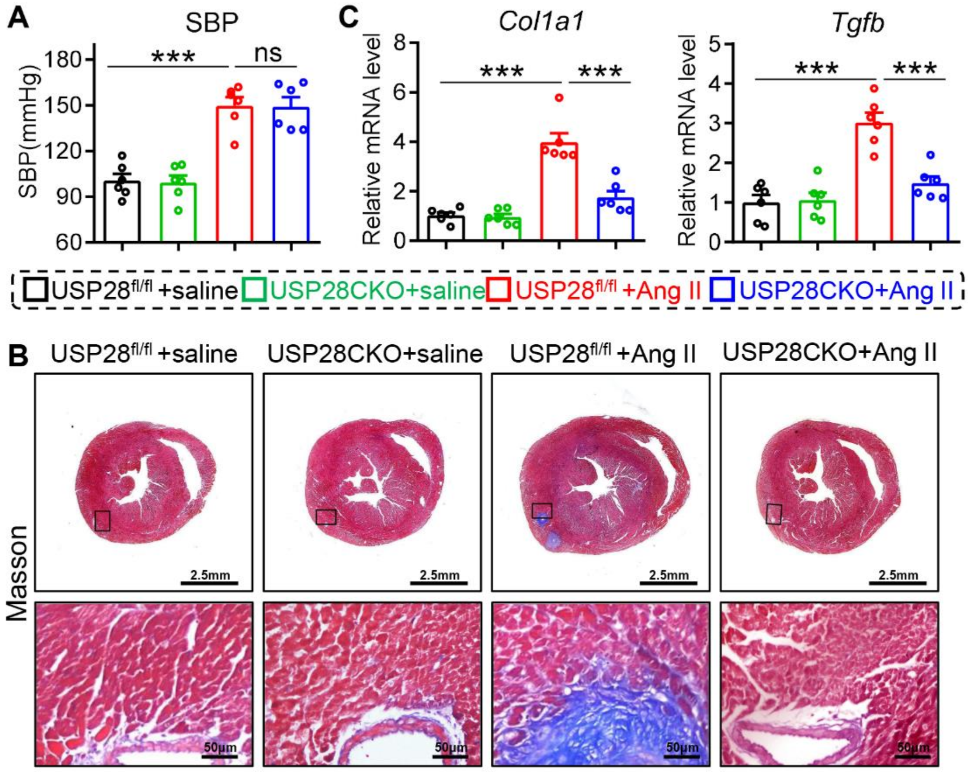

Figure S4 USP28CKO and USP28$^{fl/fl}$ mice were subcutaneously implanted with osmotic mini- pump (Saline or Ang II (1000 ng/kg/min)) for 4 weeks. **(A)** Systolic blood pressure (SBP) of indicated mice at 4 weeks (n=6). **(B)** Masson's trichome-stained sections. **(C)** The mRNA levels of *Tgfb* and *Col1a1* in heart tissues (n=7). (ns= no significant, *** P<0.001)

A

USP28 binding proteins			
	Gene names	Sequence coverage [%]	Score
1	***Usp28****	49	323.31
2	*Oasl2*	28	76.302
3	*Metap1*	51.3	68.625
4	*Micall2*	12.3	63.458
5	*Vipas39*	23.6	58.816
6	*Pabpn1*	19.2	53.508
7	*Isg15*	25.5	52.546
8	*Ruvbl2*	19.2	49.707
9	*Dnajc2*	17.1	47.03
10	*Khsrp*	12.8	41.756
11	*Cep55*	17.5	40.397
12	***Trim21***	15.3	40.145
13	*Znf622*	10.5	34.057
14	*Zc3h7a*	11.9	30.023
15	*Naf1*	16.2	28.993
16	*Arf5;Arf4;*	11.1	28.963
17	*Eif4g1*	4.8	28.506
18	*Srp9*	37.2	26.591
19	*Baiap2l1*	21.8	22.632
20	*Cand1*	11.9	22.197

*USP28 is the bait protein

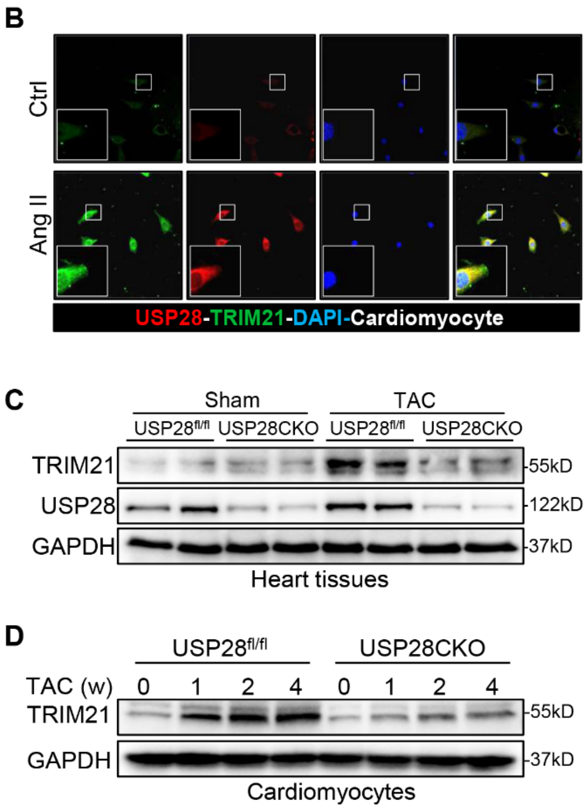

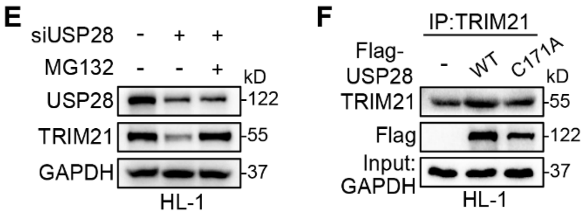

Figure S5 (A) Co-IP combined LC-MS/MS analysis of USP28 binding proteins (Top20, rank according to the USP28-IP score). **(B)** Co-localization of endogenous USP28 (red) and TRIM21 (green) in Ang II (1 µM, 24h)-stimulated HL-1. **(C)** Protein levels of TRIM21 and USP28 in heart tissues of indicated mice. **(D)** Representative western blot of TRIM21 in isolated adult mouse ventricular cardiomyocytes (AVCMs) of USP28CKO and USP28$^{fl/fl}$ mice. **(E)** HL-1 cells were transfected with siRNAs of NC (negative control) or USP28 followed by MG132 (20µM, 6-8h). Protein levels of USP28 and TRIM21 were detected. **(F)** Co-IP of Flag-USP28 and TRIM21 in lysates from HL-1 expressing Flag-USP28 (WT or C171A).

A

Gsea.enrichment.GO (USP28^{OE}+Ang II vs. EV+Ang II)			
**NAME**	**SIZE**	**NES**	**FDR.qval**
cellular response to interferon beta	47	-2.001	0.00000
cardiac muscle contraction	46	-1.888	0.00000
regulation of the force of heart contraction	20	-1.840	0.00008
heart contraction	18	-1.790	0.00042
**oxidoreductase activity, as one donor**	39	-1.722	0.00218
**monooxygenase activity**	101	-1.715	0.00245
cardiac muscle hypertrophy in response to stress	16	-1.697	0.00369
regulation of cardiac muscle cell contraction	17	-1.685	0.00483
**oxidoreductase activity**	88	-1.660	0.00699
**cytochrome c oxidase activity**	24	-1.628	0.01172
**peroxidase activity**	32	-1.610	0.01486
**phosphorus oxygen lyase activity**	21	-1.606	0.01531
**epoxygenase p450 pathway**	17	-1.599	0.01664
**antioxidant activity**	26	-1.589	0.01911
regulation of cardiac muscle contraction	20	-1.585	0.01996
ventricular cardiac muscle cell action potential	17	-1.574	0.02310

B

Gsea.enrichment.Reactome (USP28^{OE}+Ang II vs. EV+Ang II)			
**NAME**	**SIZE**	**NES**	**FDR.qval**
r mmu 397014 muscle contraction	171	-1.787	0.00014
r mmu 913531 interferon signaling	62	-1.763	0.00039
r mmu 5576891 cardiac conduction	113	-1.761	0.00038
**r mmu 211859 biological oxidations**	193	-1.715	0.00129
**r mmu 5668599 rho gtpases activate nadph oxidases**	22	-1.543	0.02834
**r mmu 3299685 detoxification of reactive oxygen species**	33	-1.348	0.19032
**r mmu 2559580 oxidative stress induced senescence**	104	-0.785	0.96905

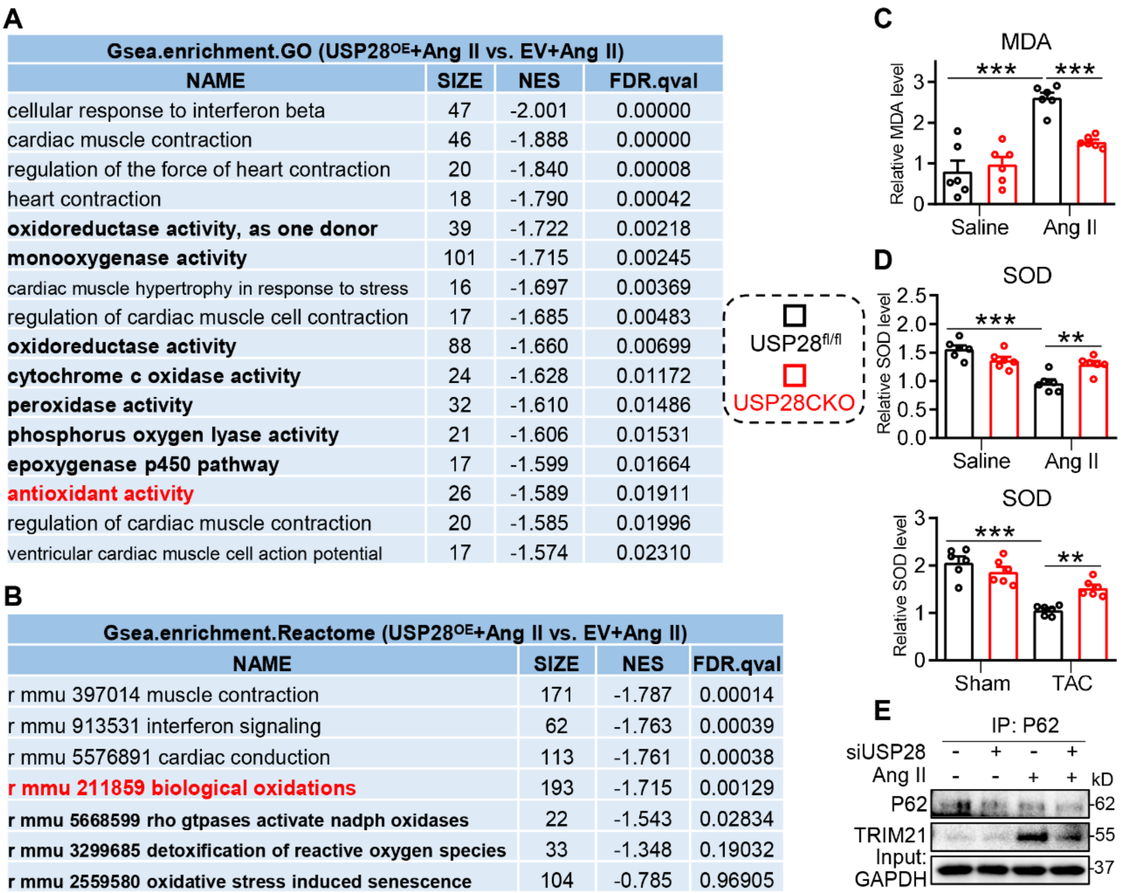

Figure S6 (A-B) GSEA enrichment analysis (**A** for GO, **B** for Reactome) of transcriptome in Ang II (1μM, 24h)-induced HL-1 cells expressing Flag-USP28 (USP28^{OE}) or EV (NES: normalized enrichment score; FDR: false discovery rate). **(C-D)** USP28CKO and USP28$^{fl/fl}$ mice were subjected to Ang II or TAC for 4 weeks. The levels of MDA and SOD in heart tissues (n=6; ** P<0.001, *** P<0.001). **(E)** HL-1 cells were transfected with siRNAs of NC (negative control) or USP28 followed by Ang II (1 μM, 24h). Co-IP was performed with anti-p62 and followed by western blot of TRIM21 and p62.

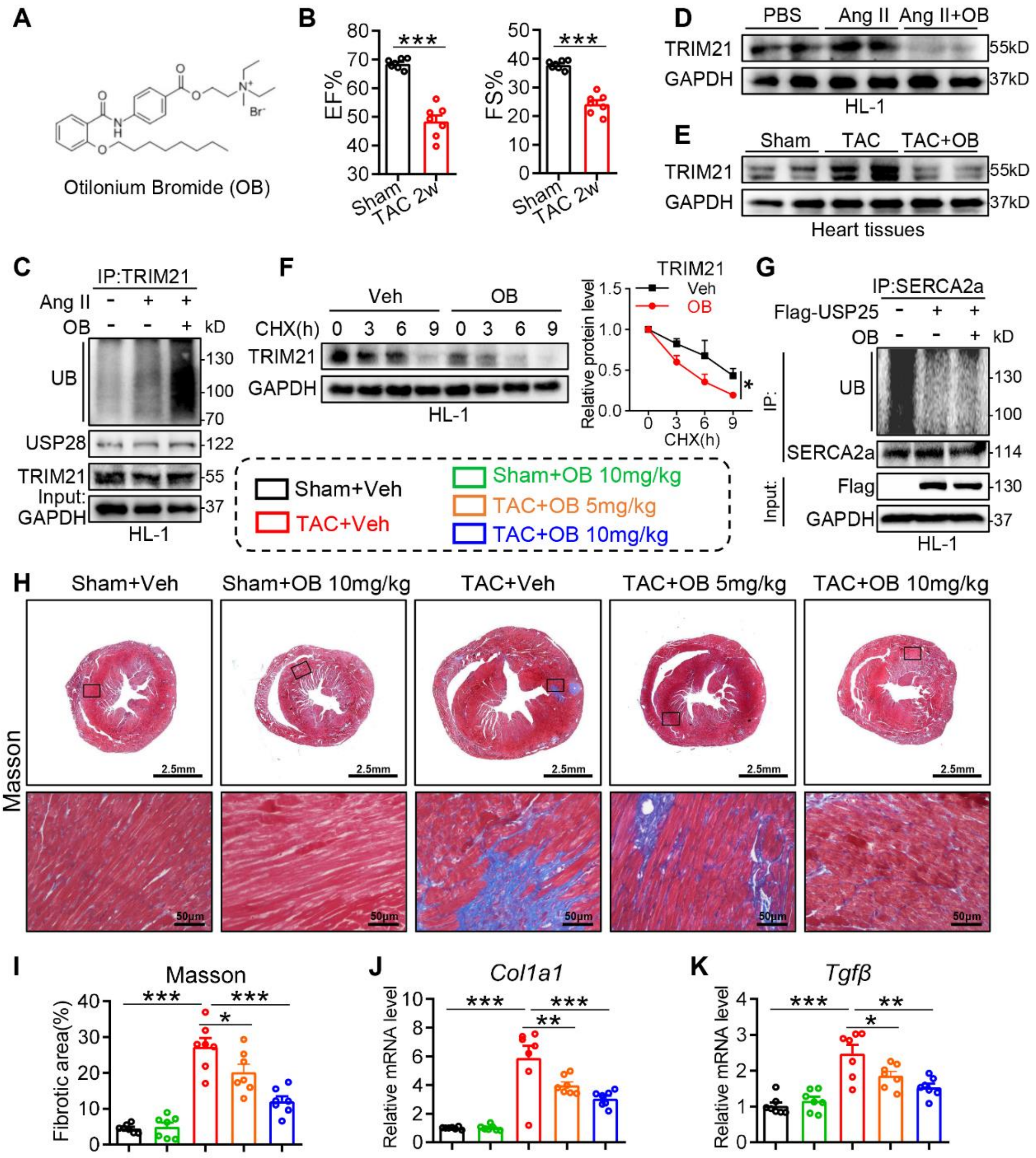

Figure S7 (A) Otilonium bromide chemical structure. **(B)** LV ejection fraction (EF) and fractional shortening (FS) of mice with TAC 2 weeks. **C-D:** HL-1 were pretreated with OB (10 μg/ml, 1h), and followed by the Ang II stimulation (1μM, 24h). **(C)** Ubiquitinated TRIM21 was enriched with anti-TRIM21 and then was detected with UB and TRIM21. **(D)** Protein level of TRIM21 in HL-1. **(E)** Protein level of TRIM21 in heart tissues of indicated mice. **(F)** Protein level of TRIM21 in HL-1 pretreated with OB (10 μg/ml, 1h) and followed by CHX (25μg/mL) pulse-chase stimulation (left) and the quantitative analysis of TRIM21 (right, n=3, * P<0.05). **(G)** Flag-USP25 were transfected into HL-1 and followed by OB (10 μg/ml, 1h). Ubiquitinated SERCA2a was enriched with anti-SERCA2a and then was detected with UB, Flag-USP25 and SERCA2a. **H-K:** Mice of indicated group were underwent TAC for 6 weeks. Selective USP28 inhibitor OB (5 or 10 mg/kg/day) was intragastric administrated at the end of 2nd week after TAC. All groups were harvested 6 weeks after TAC. **(H-I)** Representative Masson's trichome stained images of heart sections and corresponding quantitative analysis. **(J-K)** The mRNA levels of *Col1a1* and *Tgfb* in heart tissues of indicated mice. (n=7 for each group; * P<0.05, ** P<0.01, *** P<0.001)

Supplementary Tables:

Table S1: Sequences of primers for qPCR used in the study.

**Gene**	**Species**	**FW**	**RW**
Usp28	Mouse	AGGTCCGATGCTTGTCTTCTGAG	AGGCTGCTTCTACACCACTCTTC
Trim21	Mouse	GATTCACGCAGAGTTCGCACTTC	GCCAGCTCAGCCTCCTTCTTC
Myh7	Mouse	CAGAACACCAGCCTCATCAACCAG	TTCTCCTCTGCGTTCCTACACTCC
Nppa	Mouse	AAGAACCTGCTAGACCACCTGGAG	TGCTTCCTCAGTCTGCTCACTCAG
Tgfb	Mouse	TGACGTCACTGGAGTTGTACGG	TGACGTCACTGGAGTTGTACGG
Col1a1	Mouse	TGGCCTTGGAGGAAACTTTG	TGGCCTTGGAGGAAACTTTG
Nrf2	Mouse	TTTTCCATTCCCGAATTACAGT	AGGAGATCGATGAGTAAAAATGGT
Nqo1	Mouse	AGGGTTCGGTATTACGATCC	AGTACAATCAGGGCTCTTCTCG
β-actin	Mouse	CCGTGAAAAGATGACCCAGA	TACGACCAGAGGCATACAG
Nrf2	Rat	ACTGTCCCCAGCCCAGAGGC	CCAGGCGGTGGGTCTCCGTA
Nqo1	Rat	ACCTTGCTTTCCATCACCAC	CAAAGGCGAAAACTGAAAGC
β-actin	Rat	CCTAGACTTCGAGCAAGAGA	GGAAGGAAGGCTGGAAGA
Usp28	Human	GTCCTCCACCAACTCCTCATCAC	TCAGACGACAAGCAGCGAACC
β-actin	Human	CAGATGTGGATCAGCAAGCAGGAG	CGCAACTAAGTCATAGTCCGCCTAG

Table S2: Echocardiographic parameters in TAC model mice

Parameter	Sham		TAC	
	USP28$^{f/f}$	USP28CKO	USP28$^{f/f}$	USP28CKO
IVSd (mm)	0.73±0.02	0.69±0.03	0.91±0.02*	0.77±0.05$^{\#}$
IVSs (mm)	0.98±0.06	1.01±0.07	1.25±0.07*	1.14±0.03^{NS}
LVAWd (mm)	0.80±0.12	0.81±0.06	1.03±0.04^{ns}	0.93±0.05^{NS}
LVAWs (mm)	1.17±0.09	1.18±0.07	1.35±0.05^{ns}	1.25±0.06^{NS}
LVIDd (mm)	3.57±0.14	3.55±0.09	3.90±0.04^{ns}	3.43±0.16$^{\#}$
LVIDs (mm)	2.35±0.13	2.34±0.20	2.85±0.05*	2.54±0.15^{NS}
Heart rate (bpm)	416.8±21.0	433.7±20.3	421.7±16.4^{ns}	416.2±16.3^{NS}

IVS, interventricular septum; LVAW, left ventricular anterior wall; LVID, left ventricular internal diameter; d, diastole; s, systole. ns, represents p>0.05 vs WT+ Sham; *, p<0.05 vs WT+ Sham. NS, represents p>0.05 vs WT+ TAC; #, p<0.05 vs WT+ TAC.

Table S3: Echocardiographic parameters in Ang II model mice

Parameter	Saline		Ang II	
	**USP28$^{f/f}$**	**USP28CKO**	**USP28$^{f/f}$**	**USP28CKO**
**IVSd (mm)**	0.71±0.05	0.69±0.05	0.86±0.04^{ns}	0.73±0.05^{NS}
**IVSs (mm)**	0.92±0.04	0.87±0.06	1.08±0.06^{ns}	1.00±0.06^{NS}
**LVAWd (mm)**	0.69±0.05	0.69±0.04	0.98±0.04*	0.76±0.04$^{\#}$
**LVAWs (mm)**	1.05±0.06	1.05±0.03	1.50±0.06*	1.15±0.04$^{\#}$
**LVIDd (mm)**	3.68±0.20	3.51±0.10	4.24±0.05*	3.83±0.09^{NS}
**LVIDs (mm)**	2.42±0.07	2.31±0.12	3.14±0.01*	2.72±0.05$^{\#}$

IVS, interventricular septum; LVAW, left ventricular anterior wall; LVID, left ventricular internal diameter; d, diastole; s, systole. ns, represents p>0.05 vs WT+ Saline; *, p<0.05 vs WT+ Saline. NS, represents p>0.05 vs WT+ Ang II; #, p<0.05 vs WT+ Ang II.

Table S4: Echocardiographic parameters in TAC model mice subjected to Otilonium Bromide

Parameter	Sham	TAC	
	Vehicle	Vehicle	Otilonium Bromide
IVSd (mm)	0.56±0.02	0.68±0.03*	0.60±0.01$^{\#}$
IVSs (mm)	0.86±0.03	0.98±0.03*	0.89±0.03^{NS}
LVAWd (mm)	0.69±0.03	1.09±0.07*	0.77±0.03$^{\#}$
LVAWs (mm)	0.96±0.07	1.45±0.08*	1.21±0.05$^{\#}$
LVIDd (mm)	3.47±0.16	4.08±0.03*	3.78±0.07^{NS}
LVIDs (mm)	2.25±0.16	3.05±0.05*	2.79±0.04^{NS}

IVS, interventricular septum; LVAW, left ventricular anterior wall; LVID, left ventricular internal diameter; d, diastole; s, systole. ns, represents p>0.05 vs Sham+ Vehicle; *, p<0.05 vs Sham+ Vehicle. NS, represents p>0.05 vs TAC+ Vehicle; #, p<0.05 vs TAC+ Vehicle.
